# Gene Expression Profiles in Human and Mouse Primary Cells Provide New Insights into the Differential Actions of Vitamin D_3_ Metabolites

**DOI:** 10.1371/journal.pone.0075338

**Published:** 2013-10-08

**Authors:** Pentti Tuohimaa, Jing-Huan Wang, Sofia Khan, Marianne Kuuslahti, Kui Qian, Tommi Manninen, Petri Auvinen, Mauno Vihinen, Yan-Ru Lou

**Affiliations:** 1 Department of Anatomy, Medical School, University of Tampere, Tampere, Finland; 2 Department of Clinical Chemistry, Tampere University Hospital, University of Tampere, Tampere, Finland; 3 Tampere Graduate School in Biomedicine and Biotechnology, University of Tampere, Tampere, Finland; 4 Drug Discovery Graduate School, University of Turku, Turku, Finland; 5 Institute of Biomedical Technology and BioMediTech, University of Tampere, Tampere, Finland; 6 Department of Obstetrics and Gynecology, University of Helsinki and Helsinki University Central Hospital, Helsinki, Finland; 7 Institute of Biotechnology, University of Helsinki, Helsinki, Finland; 8 Department of Cell Biology, Medical School, University of Tampere, Tampere, Finland; 9 Institute of Experimental Medical Science, Lund University, Lund, Sweden; 10 Tampere University Hospital, Tampere, Finland; 11 Division of Biopharmaceutics and Pharmacokinetics, Faculty of Pharmacy, University of Helsinki, Helsinki, Finland; University of Tennessee, United States of America

## Abstract

1α,25-Dihydroxyvitamin D_3_ (1α,25(OH)_2_D_3_) had earlier been regarded as the only active hormone. The newly identified actions of 25-hydroxyvitamin D_3_ (25(OH)D_3_) and 24R,25-dihydroxyvitamin D_3_ (24R,25(OH)_2_D_3_) broadened the vitamin D_3_ endocrine system, however, the current data are fragmented and a systematic understanding is lacking. Here we performed the first systematic study of global gene expression to clarify their similarities and differences. Three metabolites at physiologically comparable levels were utilized to treat human and mouse fibroblasts prior to DNA microarray analyses. Human primary prostate stromal P29SN cells (hP29SN), which convert 25(OH)D_3_ into 1α,25(OH)_2_D_3_ by 1α-hydroxylase (encoded by the gene *CYP27B1*), displayed regulation of 164, 171, and 175 genes by treatment with 1α,25(OH)_2_D_3_, 25(OH)D_3_, and 24R,25(OH)_2_D_3_, respectively. Mouse primary *Cyp27b1* knockout fibroblasts (m*Cyp27b1* ^−/−^), which lack 1α-hydroxylation, displayed regulation of 619, 469, and 66 genes using the same respective treatments. The number of shared genes regulated by two metabolites is much lower in hP29SN than in m*Cyp27b1* ^−/−^. By using DAVID Functional Annotation Bioinformatics Microarray Analysis tools and Ingenuity Pathways Analysis, we identified the agonistic regulation of calcium homeostasis and bone remodeling between 1α,25(OH)_2_D_3_ and 25(OH)D_3_ and unique non-classical actions of each metabolite in physiological and pathological processes, including cell cycle, keratinocyte differentiation, amyotrophic lateral sclerosis signaling, gene transcription, immunomodulation, epigenetics, cell differentiation, and membrane protein expression. In conclusion, there are three distinct vitamin D_3_ hormones with clearly different biological activities. This study presents a new conceptual insight into the vitamin D_3_ endocrine system, which may guide the strategic use of vitamin D_3_ in disease prevention and treatment.

## Introduction

Vitamin D_3_ produced in the skin undergoes two sequential hydroxylation steps, first 25-hydroxylation generating 25-hydroxyvitamin D_3_ (25(OH)D_3_) and then 1α-hydroxylation generating 1α,25-dihydroxyvitamin D_3_ (1α,25(OH)_2_D_3_). Both of them are further metabolized by 24-hydroxylase. Among many endogenous vitamin D_3_ metabolites, 25(OH)D_3_ and 24R,25-dihydroxyvitamin D_3_ (24R,25(OH)_2_D_3_) are the first and second most abundant circulating metabolites of vitamin D_3_. 1α,25(OH)_2_D_3_ is believed to be the most active vitamin D_3_ metabolite. Their classic actions in calcium homeostasis and non-classical actions in cell proliferation and differentiation have been the focus of vitamin D_3_ research.

During the past two decades, there have been some important discoveries of vitamin D_3_ metabolism and endocrine system. Cells can uptake 25(OH)D_3_ by megalin-mediated endocytosis [1], [2]. 25(OH)D_3_ possesses an inherent hormonal activity regulating cell proliferation and gene expression, which was first evidenced in human prostate cells [3] and later in other types of human and mouse cells by *in vitro* as well as *in vivo* studies [4]–[13]. 25(OH)D_3_ acts synergistically with 1α,25(OH)_2_D_3_ [4]. 24R,25(OH)_2_D_3_ seems to regulate specifically chondrocytes [14] and osteogenesis [15]. 25(OH)D_3_ 1α-hydroxylase, encoded by the gene *CYP27B1* and responsible for the conversion of 25(OH)D_3_ to 1α,25(OH)_2_D_3_, is expressed in the kidney and in many extra-renal tissues [16]–[18], thus the intracellularly synthesized 1α,25(OH)_2_D_3_ was suggested to act as an intracrine factor [9]. Epidemiological studies provide accumulating evidence showing that vitamin D_3_ insufficiency is associated with infection [19], cancers [20], fractures [21], diabetes [22], and neuropsychiatric disease [23], and 25(OH)D_3_ concentration gradient is related to the seasonal variation of cancer survival [24]. The optimal dose and serum concentration of vitamin D_3_ is under debate. Some epidemiological studies also suggest that both low and high 25(OH)D_3_ levels are harmful [9], [25], [26]. Based on these findings, it seems that the vitamin D_3_ system is more complex than earlier thought.

To identify target genes, microarray gene expression studies have been performed in various cellular systems after treatment with 1α,25(OH)_2_D_3_, reviewed by C. Kriebitzsch, et al [27]. Gene expression in response to 25(OH)D_3_ or 24R,25(OH)_2_D_3_ has not yet been studied by DNA microarray. Here we compared the effects of 1α,25(OH)_2_D_3_, 25(OH)D_3_, and 24R,25(OH)_2_D_3_ on gene expression patterns to clarify similarities and differences in signal transduction. To exclude the effect of the intracellular product of 1α,25(OH)_2_D_3_, we also performed microarray study in mouse primary *Cyp27b1* knockout fibroblasts (m*Cyp27b1* ^−/−^). To our knowledge, microarray gene expression studies have not been made in m*Cyp27b1* ^−/−^ cells. Our data show that there are three vitamin D_3_ (cholecalcipherol) hormonal systems, which display partially independent gene transcriptional effects.

## Materials and Methods

### Ethics Statement

All the animal experiments were approved by the Ethical Committee of the University of Tampere. Animal care and experimental procedures were conducted in accordance with the European legislation and the guidelines of the Federation of European Laboratory Animal Associations.

### Mice

*Cyp27b1* knockout (*Cyp27b1* ^−/−^) mice were bred at the University of Tampere from the line initially generated at the Shriners Hospital, Montreal, Canada [28]. Feeding and genotyping *Cyp27b1* ^−/−^ mice were described previously [4].

### Cell Culture, Vitamin D_3_ Treatment, and RNA Isolation

The isolation and characterization of human primary prostate stromal P29SN cells were described previously [3]. The vast majority of the primary stromal culture was shown to be fibroblasts in phenotype. Over 99% of the cells were positive for fibroblast markers vimentin and fibronectin. Less than 5% of the cells were positive for smooth muscle actin and less than 2% were positive for desmin [3]. The cells were maintained in phenol red-free DMEM/F12 medium (Sigma-Aldrich, Steinheim, Germany), supplemented with 10% fetal bovine serum (FBS), 3 mM L-glutamine, 5 µg/ml insulin, and antibiotics (penicillin 100 units/ml, streptomycin 100 µg/ml) (Gibco-BRL, Life Technology, Paisley, Scotland) at 37°C in a humid atmosphere with 5% CO_2_. 24 hours prior to treatments, growth media containing 10% FBS-DCC (10% FBS treated with dextran coated charcoal) were added to semi-confluent hP29SN cells. Next day the cells were treated with 0.1% ethanol, 10 nM 1α,25(OH)_2_D_3_, 500 nM 25(OH)D_3_, or 25 nM 24R,25(OH)_2_D_3_ (kindly provided by Leo Pharmaceuticals, Ballerup, Denmark) for 24 hours. Ethanol concentration was 0.1% in all treatment conditions. Experiments were replicated four independent times for each treatment condition.

The isolation and culture of mouse primary *Cyp27b1* knockout skin fibroblasts (m*Cyp27b1* ^−/−^ fibroblasts) were described previously [4]. 48 hours prior to experiments, growth media were supplemented with 10% FBS-DCC. Fibroblasts were treated with 0.1% ethanol, 10 nM 1α,25(OH)_2_D_3_, 500 nM 25(OH)D_3_, or 50 nM 24R,25(OH)_2_D_3_ for 24 hours. Ethanol concentration was 0.1% in all treatment conditions. Experiments were replicated four independent times for each treatment condition.

Total cellular RNA was isolated from hP29SN stromal cells by TRIzol reagent (Invitrogen, Carlsbad, CA, USA) and from m*Cyp27b1* ^−/−^ fibroblasts by RNeasy Mini Kit (Qiagen GmbH, Hilder, Germany) following the manufacturers’ protocols. The RNA concentration and purity were verified using a GeneQuant II (Pharmacia Biotech, Piscataway, NJ, USA). The A280/A260 ratio of all RNA samples utilized for experimentation was 1.9–2.1. Randomly selected RNA samples were subjected to denaturing-gel electrophoresis. The ratio of the intensity of 28S and 18S bands was 1.5–2.0.

### Quantitative Real-time RT-PCR (qRT-PCR)

*CYP24A1* (encoding vitamin D_3_ 24-hydroxylase) gene expression was measured by using qRT-PCR to ensure that hP29SN stromal cells were successfully stimulated by vitamin D_3_ metabolites. Similarly, for the validation of microarray data, the expression levels of eight differentially expressed genes in hP29SN stromal cells and two genes in m*Cyp27b1* ^−/−^ fibroblasts were analyzed. The qRT-PCR analysis was performed as previously described [3]. Briefly, total RNA was converted to cDNA by using High Capacity Archive Kit and the 20 ng of cDNAs were used in real time qPCR reactions by using SYBR Green PCR Master Mix kit (Applied Biosystems, Foster City, CA, USA). Primers were designed by using Primer Express v2.0 software (Applied Biosystems, Foster City, CA, USA). The primer sequences are listed in Table S1. The expression levels of target genes were normalized against the housekeeping gene acidic ribosomal phosphoprotein P0 (*RPLP0*) in hP29SN stromal cells and TATA box binding protein (*Tbp*) in m*Cyp27b1* ^−/−^ fibroblasts.

### Affymetrix Microarray Analysis

cRNA preparation, hybridization, and the array wash procedure were performed following the standard protocol from Affymetrix. In brief, 7 µg of total RNA from each hP29SN sample were used to synthesize double-stranded cDNA. The cDNA was used as a template to generate biotinylated cRNA by an *in vitro* transcription reaction. 20 µg of biotinylated cRNA was fragmented and added to the GeneChip® Human Genome U133 Plus 2.0 Arrays (Affymetrix, Santa Clara, CA, USA). The hybridization was carried out at 45°C for 20 hours with a rotation at 60 rpm in an Affymetrix hybridization oven, and then the arrays were washed and stained with a streptavidin-conjugated fluorescent stain followed by antibody amplification on the Affymetrix Fluidics Station 400. Scanned images were processed using Affymetrix GeneChip® Operating Software Server 1.0 (GCOS Server) (Affymetrix, Santa Clara, CA, USA).

For mouse RNA samples, similar procedures were followed except for the following: 250 ng of total RNA from each m*Cyp27b1* ^−/−^ sample was used as a starting material. Each sample was hybridized to GeneChip® Mouse Genome 430 2.0 Arrays (Affymetrix, Santa Clara, CA, USA) at 45°C for 16 hours according to 3′ IVT Express Kit User Manual (Affymetrix, Santa Clara, CA, USA). GeneChip® Fluidics Station 450 was used to wash and stain the arrays. GeneChip® Scanner 3000 7G with AutoLoader was used to scan the arrays.

### Array Data Analysis

Data from array scans were normalized and analyzed by GeneSpring GX 7.3.1 Expression Analysis software (Agilent Technologies, Santa Clara, CA, USA). Before normalization, expression values less than 0.01 were converted to 0.01 to enable more efficient analysis of log-transformed data. To control chip-wide variation in intensity values, each chip was normalized to the 50^th^ percentile of all measurements (per-chip normalization). As per gene normalization, treatment samples were normalized against the median of control samples. Each measurement for each gene in the treatment samples was divided by the median of that gene’s measurements in the corresponding control samples. In all three conditions, the present call was demanded only to respective conditions. We used Pearson Correlation as similarity measure and unsupervised average linkage hierarchical clustering algorithm. The program was asked to merge similar branches with separation ratio of one and minimum distance of 0.001 and to calculate confidence levels (bootstrapping) with 100 datasets.

The microarray data has been deposited to ArrayExpress with the accession numbers of E-MTAB-1773 for human data and E-MTAB-1774 for mouse data.

### Functional Annotation, Network Generation and Pathway Analyses

The DAVID Functional Annotation Bioinformatics Microarray Analysis tools (http://david.abcc.ncifcrf.gov/) [29], [30] were used to study the biological meaning of regulated genes. Functional properties of differentially expressed genes were further analyzed in the context of Gene Ontology (GO) and molecular networks using the Ingenuity Pathways Analysis (IPA; Ingenuity Systems®; http://www.ingenuity.com). The number of molecules in the network was limited to default of 35, based on the number of connections between the input genes and genes in the IPA database. For each of the network, a score is calculated by the IPA according to the fit of that network to the input genes. The score (derived from a *p*-value) represents the likelihood of random change. For example, a score of 2 indicates that there is a 1% change that the input genes are together in a network due to random change. Therefore, scores of 2 or higher have at least a 99% confidence of not being generated by random change alone. The highest scoring networks were selected.

## Results

### Gene Expression Profiles Induced by Vitamin D_3_ Metabolites

We aimed at exploring the gene expression profiles of three vitamin D_3_ metabolites using hP29SN stromal cells and m*Cyp27b1* ^−/−^ fibroblasts. Human P29SN stromal cells were derived from a normal area of human prostatic carcinoma [3]. Mouse *Cyp27b1* ^−/−^ fibroblasts were derived from *Cyp27b1* ^−/−^ mice that do not express 25(OH)D_3_ 1α-hydroxylase and therefore cannot convert 25(OH)D_3_ into 1α,25(OH)_2_D_3_.

We verified the responsiveness and successful stimulation of cells by vitamin D_3_ metabolites by measuring mRNA level of *CYP24A1* in each sample by qRT-PCR before applying samples to microarray assays (Figure S1). RNA samples of corresponding metabolite treatments from randomly selected two sets of experiments were pooled to generate final two sets of RNA for microarray hybridizations.

**Figure 1:**
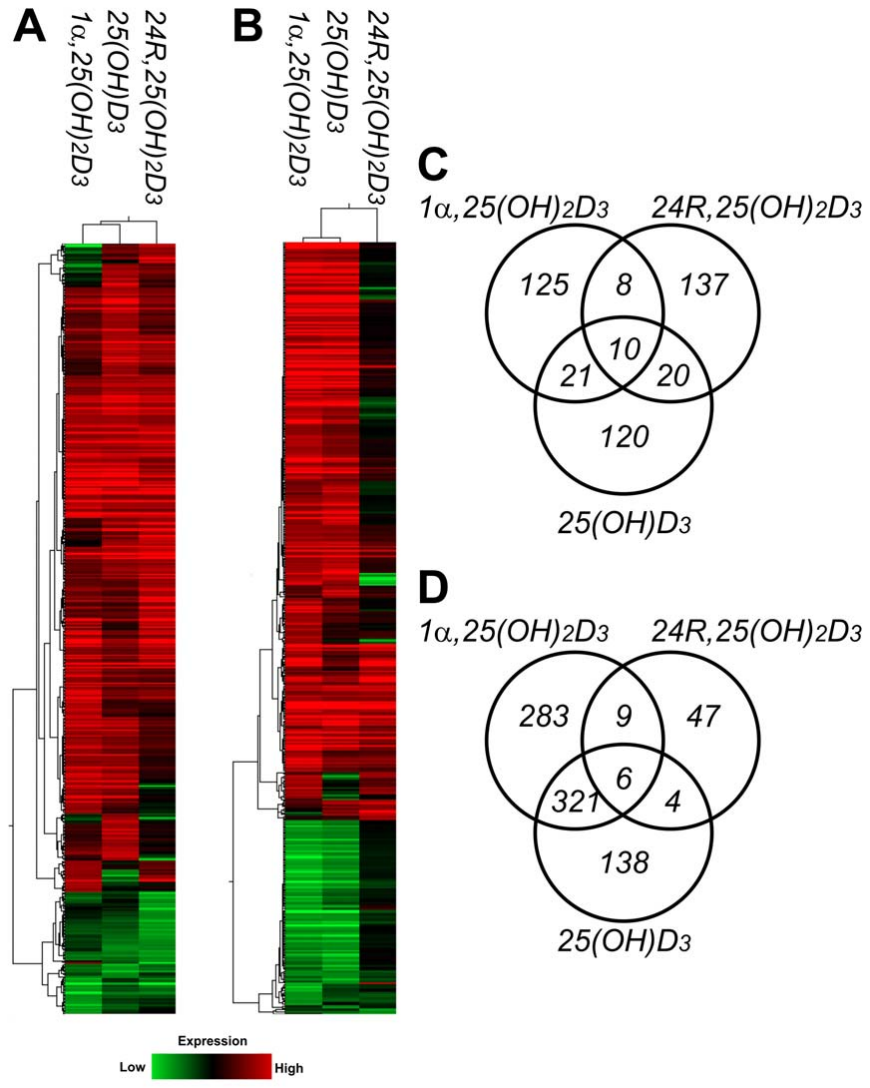
Gene expression profiles. Hierarchical clustering of the differentially expressed genes in (A) hP29SN stromal cells and (B) m*Cyp27b1* ^−/−^ fibroblasts after vitamin D_3_ treatments. Colored-bands represent the change of the corresponding gene expression, green indicating down-regulation and red up-regulation. The key for deciphering of the color is shown below the clustering image. Venn diagrams of co-expressed and uniquely regulated genes by vitamin D_3_ metabolites in (C) hP29SN and (D) m*Cyp27b1* ^−/−^ fibroblasts.

The gene expression profiles in hP29SN stromal cells treated with 10 nM 1α,25(OH)_2_D_3_, 500 nM 25(OH)D_3_, or 25 nM 24R,25(OH)_2_D_3_ were determined by GeneChip® Human Genome U133 Plus 2.0 Arrays that contained more than 54000 probe sets to analyze the expression level of more than 47000 transcripts and variants, including approximately 38500 well-characterized human genes. Ethanol-treated samples served as negative control. Each vitamin D_3_ metabolite-treated sample was compared with ethanol-treated samples. Only those genes that exhibited at least twofold change in gene expression in parallel experiments were reported to ensure the fidelity of the data. The final result was the average of the two independent microarray experiments. For 1α,25(OH)_2_D_3_ treatment, 164 genes met the selection criteria while 171 and 175 genes were identified for 25(OH)D_3_ and 24R,25(OH)_2_D_3_ treatment, respectively (data not shown). All the genes that displayed at least twofold expression change in any of the treatments were clustered using hierarchical clustering method by GeneSpring software (Figure 1A). To understand the specific role of each vitamin D_3_ metabolite, we grouped the regulated genes into commonly and uncommonly regulated gene groups (Figure 1C). Of the genes met the selection criteria, only 10 are common in all the three experimental conditions. Interestingly, all these genes were up-regulated. The number of genes significantly regulated in two conditions are 21 for 1α,25(OH)_2_D_3_ and 25OHD_3_, 8 for 1α,25(OH)_2_D_3_ and 24R,25(OH)_2_D_3_, and 20 for 25OHD_3_ and 24R,25(OH)_2_D_3_, respectively (Figure 1C).

Mouse *Cyp27b1* ^−/−^ fibroblasts were treated either with 10 nM 1α,25(OH)_2_D_3_, 500 nM 25(OH)D_3_, or 50 nM 24R,25(OH)_2_D_3_, and GeneChip® Mouse Genome 430 2.0 Arrays were used to study the gene expression profile. Using a cut-off of twofold expression change, we identified 619, 469, and 66 genes regulated by 1α,25(OH)_2_D_3_, 25(OH)D_3_, and 24R,25(OH)_2_D_3_, respectively (data not shown). Samples were grouped using hierarchical clustering method based on the overall expression changes in the samples (Figure 1B). The gene expression profile in 50 nM 24R,25(OH)_2_D_3_-treated cells displays much less changes compared with ethanol control, suggesting lower bioactivity of 24R,25(OH)_2_D_3_. The Venn diagram shows six genes commonly regulated by the three metabolites, 321 by 1α,25(OH)_2_D_3_ and 25OHD_3_, 9 by 1α,25(OH)_2_D_3_ and 24R,25(OH)_2_D_3_, and 4 by 25OHD_3_ and 24R,25(OH)_2_D_3_, respectively (Figure 1D).

### Functional Annotation of Genes Regulated by Vitamin D_3_ Metabolites

The biological relevance of the specifically regulated genes by each metabolite was further analyzed by the DAVID Functional Annotation Bioinformatics Microarray Analysis tools (http://david.abcc.ncifcrf.gov/) [29], [30]. The most enriched GO categories with *p*-value <0.05 are listed in Tables S2, S3, and S4. The enriched GO categories show clear differences between the profiles of the three metabolites, which suggests that each of them has a unique biological role.

In hP29SN stromal cells, the highly enriched GO categories related to 1α,25(OH)_2_D_3_-treatment include those involved in nucleotide biosynthesis, ubiquitin conjugation, ion binding, and macromolecule biosynthesis (Table S2). 25(OH)D_3_ plays numeral roles in intracellular organelles, DNA-binding transcription factors, protein-protein interaction, transcription coregulator activity, RNA-binding motif, and organic acid biosynthesis (Table S2). 24R,25(OH)_2_D_3_ appeared to be involved in the regulation of zinc-finger motifs, methylation, transcription, epigenetics, protein kinases, and membrane targeting (Table S2).

In m*Cyp27b1* ^−/−^ fibroblasts, 1α,25(OH)_2_D_3_ is specifically involved in cell differentiation, extracellular matrix, cell adhesion, prostate gland development, cytokine and growth factor activity, cell motion, mesenchymal and epithelial cell proliferation, neuron development, EGF, and gland development (Table S3). On the other hand, 25(OH)D_3_ plays a different role, mainly in the regulation of cell apoptosis, lipid transport, glycoprotein, extracellular matrix components, blood vessel development, cell proliferation, and response to wounding (Table S3). However, 24R,25(OH)_2_D_3_ appears to have very limited biological activity based on the current detection method, only being involved in ion binding (Table S3). We were also interested to know the common biological roles of 25OHD_3_ and 1α,25(OH)_2_D_3_, and therefore performed functional annotation analysis of 321 commonly regulated genes. These gene products are involved in glycoproteins, extracellular matrix-related proteins and enzymes, EGF-like calcium-binding, epithelium development, and cell morphogenesis (Table S4).

### Pathway Analyses and Network Generation of Genes Exclusively Regulated by Vitamin D_3_ Metabolites

**Figure 2:**
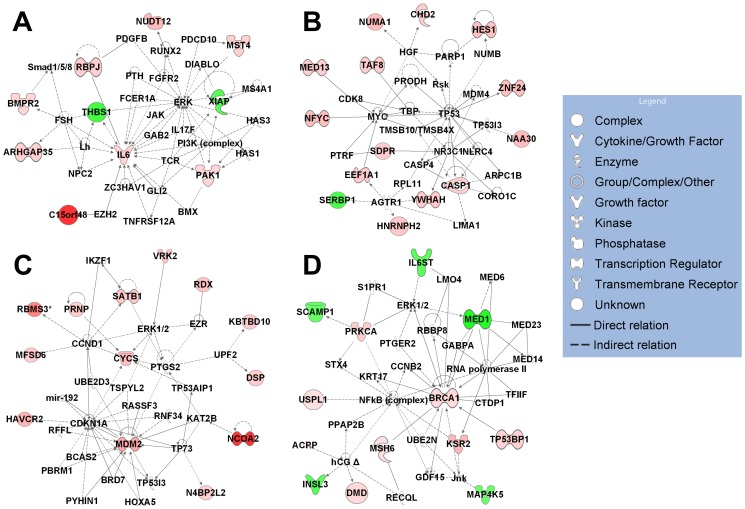
IPA diagrams of the top associated network generated for genes exclusively regulated by each metabolite in hP29SN stromal cells. (A) Genes regulated by 1α,25(OH)_2_D_3_ in Cardiovascular System Development and Function, Tissue Development, Organismal Development. (B) Genes regulated by 25(OH)D_3_ in Cell Death and Survival, Gene Expression, Tissue Morphology. (C) Genes regulated by 25(OH)D_3_ in Cell Death and Survival, Cellular Growth and Proliferation, Cell Cycle. (D) Genes regulated by 24R,25(OH)_2_D_3_ in Cell Morphology, Cellular Function and Maintenance, DNA Replication, Recombination, and Repair. Green indicates gene down-regulation and pink to red indicate gene up-regulation (the more intensive the color, the higher the expression level). An asterisk (*) indicates that multiple identifiers in the microarray set map to a single gene.

**Figure 3:**
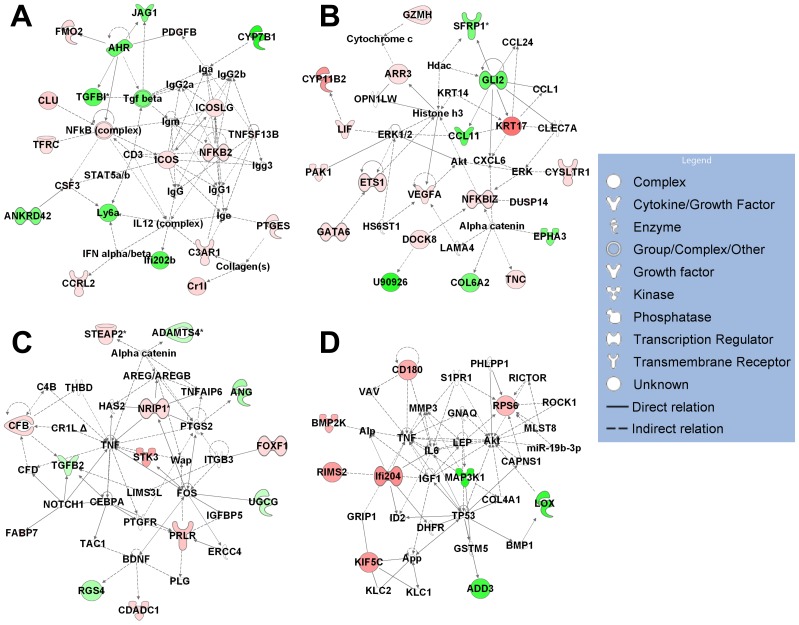
IPA diagrams of the top associated network generated for genes exclusively regulated by each metabolite in m*Cyp27b1* ^−/−^ fibroblasts. (A) Genes regulated by 1α,25(OH)_2_D_3_ in Tissue Morphology, Hematological System Development and Function, Humoral Immune Response. (B) Genes regulated by 1α,25(OH)_2_D_3_ in Cardiovascular System Development and Function, Inflammatory Response, Cellular Movement. (C) Genes regulated by 25(OH)D_3_ in Cellular Growth and Proliferation, Cellular Development, Hair and Skin Development and Function. (D) Genes regulated by 24R,25(OH)_2_D_3_ in Cell Death and Survival, Endocrine System Development and Function, Lipid Metabolism. Green indicates gene down-regulation and pink to red indicate gene up-regulation (the more intensive the color, the higher the expression level). An asterisk (*) indicates that multiple identifiers in the microarray set map to a single gene.

To reveal the possible biological interaction of specifically regulated genes by each metabolite, we performed network analyses using the IPA. The top five associated network functions of genes exclusively regulated by single metabolite are summarized in Table 1. 1α,25(OH)_2_D_3_-specific target genes are mostly involved in “Cardiovascular System Development and Function, Tissue Development, Organismal Development” (score 16, Figure 2A) and “Cellular Movement, Immune Cell Trafficking, Haematological System Development and Function” (score 14) in hP29SN stromal cells and “Tissue Morphology, Hematological System Development and Function, Humoral Immune Response” (score 28, Figure 3A) and “Cardiovascular System Development and Function, Inflammatory Response, Cellular Movement” (score 28, Figure 3B) in m*Cyp27b1* ^−/−^ fibroblasts. The most populated biological networks for 25(OH)D_3_-specific target genes are entitled “Cell Death and Survival, Gene Expression, Tissue Morphology” (score 25, Figure 2B) and “Cell Death and Survival, Cellular Growth and Proliferation, Cell Cycle” (score 23, Figure 2C) in hP29SN stromal cells and “Cellular Growth and Proliferation, Cellular Development, Hair and Skin Development and Function” (score 19, Figure 3C) in m*Cyp27b1* ^−/−^ fibroblasts. 24R,25(OH)_2_D_3_ specific target genes are mostly involved in “Cell Morphology, Cellular Function and Maintenance, DNA Replication, Recombination, and Repair” (score 22, Figure 2D) and “Gene Expression, Cellular Growth and Proliferation, Cell Cycle” (score 20) in hP29SN stromal cells and “Cell Death and Survival, Endocrine System Development and Function, Lipid Metabolism” (score 18, Figure 3D) in m*Cyp27b1* ^−/−^ fibroblasts.

**Table 1:** Top five associated network functions of genes exclusively regulated by single metabolite generated by IPA.

Human P29SN stromal cells		
Metabolite	Top associated network functions	Score
1α,25(OH)_2_D_3_	Cardiovascular System Development and Function, Tissue Development, Organismal Development	16
	Cellular Movement, Immune Cell Trafficking, Hematological System Development and Function	14
	Cardiac Proliferation, Cardiovascular System Development and Function, Cell Cycle	2
	Molecular Transport, Protein Synthesis, Protein Trafficking	2
	Antimicrobial Response, Inflammatory Response, Antigen Presentation	2
25(OH)D_3_	Cell Death and Survival, Gene Expression, Tissue Morphology	25
	Cell Death and Survival, Cellular Growth and Proliferation, Cell Cycle	23
	Cell Cycle, Cancer, Cell Death and Survival	8
	Cell Morphology, Cellular Growth and Proliferation, Cellular Assembly and Organization	2
	Cell Cycle, DNA Replication, Recombination, and Repair, Cell Death and Survival	2
24R,25(OH)_2_D_3_	Cell Morphology, Cellular Function and Maintenance, DNA Replication, Recombination, and Repair	22
	Gene Expression, Cellular Growth and Proliferation, Cell Cycle	20
	Cellular Growth and Proliferation, Cellular Development, Cellular Movement	18
	Cancer, Cell Death, Neurological Disease	2
	Cell Death, Cellular Development, Digestive System Development and Function	2
**Mouse ** ***Cyp27b1*** **^−/−^ fibroblasts**		
**Metabolite**	**Top associated network functions**	**Score**
1α,25(OH)_2_D_3_	Tissue Morphology, Hematological System Development and Function, Humoral Immune Response	28
	Cardiovascular System Development and Function, Inflammatory Response, Cellular Movement	28
	Cancer, Respiratory Disease, Carbohydrate Metabolism	22
	Organismal Injury and Abnormalities, Cell Morphology, Lipid Metabolism	16
	Cellular Growth and Proliferation, Cell Death and Survival, Cell Cycle	16
25(OH)D_3_	Cellular Growth and Proliferation, Cellular Development, Hair and Skin Development and Function	19
	Cellular Function and Maintenance, Cell-mediated Immune Response, Cellular Development	17
	Cellular Development, Cellular Growth and Proliferation, Hematological System Development and Function	15
	Gene Expression, Developmental Disorder, Cancer	15
	Cell Death, Cancer, Cell Cycle	13
24R,25(OH)_2_D_3_	Cell Death and Survival, Endocrine System Development and Function, Lipid Metabolism	18
	Connective Tissue Development and Function, Connective Tissue Disorders, Developmental Disorder	2
	Developmental Disorder, Immunological Disease, Cell Cycle	2
	Hereditary Disorder, Metabolic Disease, Cancer	2
	Cancer, Cell Death and Survival, Cellular Compromise	2

Furthermore, the top five canonical pathways for genes exclusively regulated by a single metabolite are listed in Table 2. The top canonical pathway in hP29SN stromal cells affected by 1α,25(OH)_2_D_3_, 25(OH)D_3_, and 24R,25(OH)_2_D_3_ is “Amyotrophic Lateral Sclerosis Signaling” (*p* = 0.006), “Granzyme B Signaling” (*p* = 0.002), and “DNA Double-Strand Break Repair by Homologous Recombination” (*p* = 0.003), respectively. The most affected pathway in m*Cyp27b1* ^−/−^ fibroblasts by 1α,25(OH)_2_D_3_, 25(OH)D_3_, and 24R,25(OH)_2_D_3_ is “Xenobiotic Metabolism Signaling” (*p* = 0.005), “Aryl Hydrocarbon Receptor Signaling” (*p* = 0.003), and “Production of Nitric Oxide and Reactive Oxygen Species in Macrophages” (*p* = 0.005), respectively.

**Table 2:** Top five canonical pathways generated by IPA constructed from genes exclusively regulated by single metabolite.

Human P29SN stromal cells			
Metabolite	Top canonical pathways	*p*-value	Ratio
1α,25(OH)_2_D_3_	Amyotrophic Lateral Sclerosis Signaling	6.31E-03	3/100 (0.03)
	TNFR1 Signaling 2/51	1.43E-02	2/51 (0.039)
	Xenobiotic Metabolism Signaling	1.51E-02	4/262 (0.015)
	PXR/RXR Activation 2/64	2.35E-02	2/64 (0.031)
	RhoGDI Signaling	2.92E-02	3/188 (0.016)
25(OH)D_3_	Granzyme B Signaling	2.32E-03	2/16 (0.125)
	Cell Cycle: G2/M DNA Damage Checkpoint Regulation	1.61E-02	2/48 (0.042)
	Aryl Hydrocarbon Receptor Signaling	2.14E-02	3/141 (0.021)
	Myc Mediated Apoptosis Signaling	2.83E-02	2/60 (0.033)
	Cell Cycle: G1/S Checkpoint Regulation	2.92E-02	2/64 (0.031)
24R,25(OH)_2_D_3_	DNA Double-Strand Break Repair by Homologous Recombination	2.58E-03	2/16 (0.125)
	ATM Signaling	3.73E-03	3/59 (0.051)
	Complement System	1.32E-02	2/33 (0.061)
	Role of BRCA1 in DNA Damage Response	4.27E-02	2/63 (0.032)
	Melatonin Signaling	5.63E-02	2/71 (0.028)
**Mouse ** ***Cyp27b1*** **^−/−^ fibroblasts**			
**Metabolite**	**Top canonical pathways**	***p*** **-value**	**Ratio**
1α,25(OH)_2_D_3_	Xenobiotic Metabolism Signaling	4.62E-03	9/263 (0.034)
	Ephrin Receptor Signaling	7.42E-03	7/184 (0.037)
	Hepatic Fibrosis/Hepatic Stellate Cell Activation	9.24E-03	6/138 (0.043)
	Renal Cell Carcinoma Signaling	1.17E-02	4/69 (0.058)
	Lipoate Biosynthesis and Incorporation II	2.66E-02	1/2 (0.5)
25(OH)D_3_	Aryl Hydrocarbon Receptor Signaling	2.59E-03	5/139 (0.036)
	Metabolism of Xenobiotics by Cytochrome P450	3.15E-03	4/93 (0.043)
	LPS/IL-1 Mediated Inhibition of RXR Function	1.32E-02	5/214 (0.023)
	Xenobiotic Metabolism Signaling	2.96E-02	5/264 (0.019)
	Arachidonic Acid Metabolism	4.34E-02	3/110 (-0.027)
24R,25(OH)_2_D_3_	Production of Nitric Oxide and Reactive Oxygen Species in Macrophages	4.67E-03	3/175 (0.017)
	Ceramide Signaling	1.01E-02	2/78 (0.026)
	Mineralocorticoid Biosynthesis	1.82E-02	1/10 (0.1)
	Glucocorticoid Biosynthesis	2.02E-02	1/11 (0.091)
	p70S6K Signaling	2.28E-02	2/120 (0.017)

**Figure 4:**
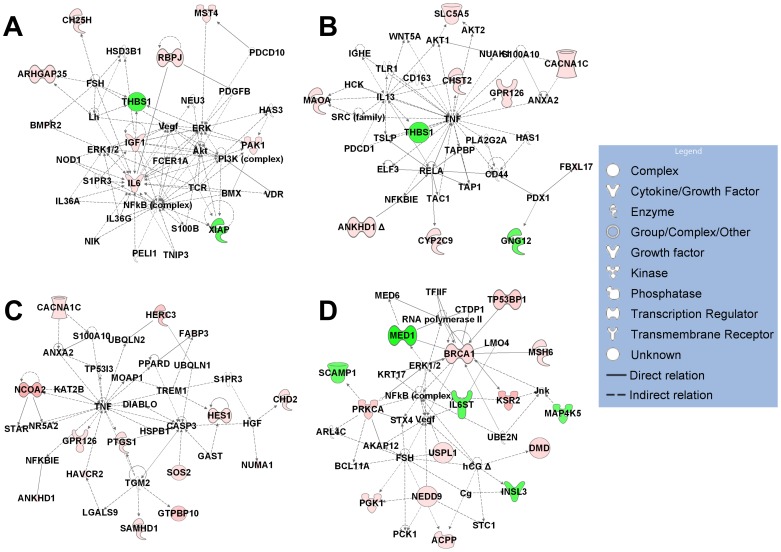
IPA diagrams of the top associated network generated for all the regulated genes in hP29SN stromal cells. (A) Genes regulated by 1α,25(OH)_2_D_3_ in Cancer, Cardiovascular System Development and Function, Cellular Movement. (B) Genes regulated by 1α,25(OH)_2_D_3_ in Cellular Movement, Hematological System Development and Function, Immune Cell Trafficking. (C) Genes regulated by 25(OH)D_3_ in Cell Death and Survival, Metabolic Disease, Endocrine System Disorders. (D) Genes regulated by 24R,25(OH)_2_D_3_ in Cellular Development, Reproductive System Development and Function, Cell Morphology. Green indicates gene down-regulation and pink to red indicate gene up-regulation (the more intensive the color, the higher the expression level).

**Figure 5:**
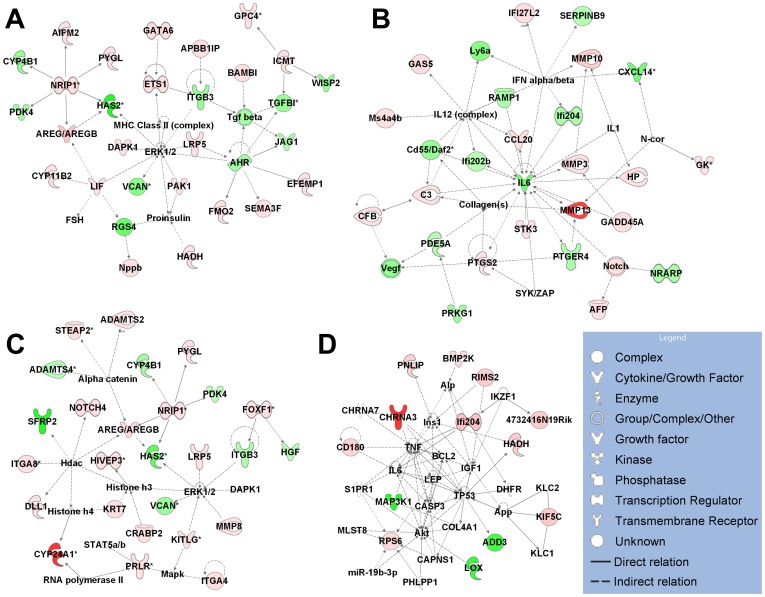
IPA diagrams of the top associated network generated for all the regulated genes in m*Cyp27b1* ^−/−^ fibroblasts. (A) Genes regulated by 1α,25(OH)_2_D_3_ in Cardiovascular System Development and Function, Organismal Development, Cancer. (B) Genes regulated by 25(OH)D_3_ in Humoral Immune Response, Inflammatory Response, Cellular Development. (C) Genes regulated by 25(OH)D_3_ in Cancer, Cellular Growth and Proliferation, Connective Tissue Disorders. (D) Genes regulated by 24R,25(OH)_2_D_3_ in Hematological System Development and Function, Hematopoiesis, Tissue Morphology. Green indicates gene down-regulation and pink to red indicate gene up-regulation (the more intensive the color, the higher the expression level). An asterisk (*) indicates that multiple identifiers in the microarray set map to a single gene.

To have an overall view of the networks affected by each metabolite, we constructed IPA network analyses using all the regulated genes in hP29SN stromal cells (Figure 4) and m*Cyp27b1* ^−/−^ fibroblasts (Figure 5). The top five associated network functions of all the genes regulated by single metabolite are summarized in Table 3.

**Table 3:** Top five associated network functions of all the genes regulated by single metabolite generated by IPA.

Human P29SN stromal cells		
Metabolite	Top associated network functions	Score
1α,25(OH)_2_D_3_	Cancer, Cardiovascular System Development and Function, Cellular Movement	15
	Cellular Movement, Hematological System Development and Function, Immune Cell Trafficking	15
	Cell Death, Cell Morphology, DNA Replication, Recombination, and Repair	13
	Hereditary Disorder, Neurological Disease, Ophthalmic Disease	2
	Cardiac Proliferation, Cardiovascular System Development and Function, Cell Cycle	2
25(OH)D_3_	Cell Death and Survival, Metabolic Disease, Endocrine System Disorders	22
	Cancer, Hematological Disease, Cellular Development	20
	Cell Cycle, Cell Death and Survival, Cellular Compromise	18
	Gene Expression, Cell Death and Survival, Amino Acid Metabolism	11
	Gene Expression, RNA Post-Transcriptional Modification, Cell-To-Cell Signaling and Interaction	2
24R,25(OH)_2_D_3_	Cellular Development, Reproductive System Development and Function, Cell Morphology	25
	Cell Cycle, Cell Death, Cellular Movement	19
	Gene Expression, DNA Replication, Recombination, and Repair, Cardiovascular System Development and Function	17
	Cell Cycle, Cellular Growth and Proliferation, DNA Replication, Recombination, and Repair	3
	Hereditary Disorder, Neurological Disease, Ophthalmic Disease	2
**Mouse ** ***Cyp27b1*** **^−/−^ fibroblasts**		
**Metabolite**	**Top associated network functions**	**Score**
1α,25(OH)_2_D_3_	Cardiovascular System Development and Function, Organismal Development, Cancer	40
	Inflammatory Response, Hematological System Development and Function, Tissue Morphology	38
	Inflammatory Response, Cellular Movement, Immune Cell Trafficking	26
	Protein Synthesis, Cellular Development, Hematological System Development and Function	21
	Dermatological Diseases and Conditions, Hereditary Disorder, Organismal Injury and Abnormalities	19
25(OH)D_3_	Humoral Immune Response, Inflammatory Response, Cellular Development	37
	Cancer, Cellular Growth and Proliferation, Connective Tissue Disorders	37
	Hematological System Development and Function, Humoral Immune Response, Tissue Morphology	25
	Organismal Injury and Abnormalities, Inflammatory Response, Connective Tissue Disorders	18
	Cellular Development, Connective Tissue Development and Function, Tissue Morphology	15
24R,25(OH)_2_D_3_	Hematological System Development and Function, Hematopoiesis, Tissue Morphology	26
	Connective Tissue Development and Function, Connective Tissue Disorders, Developmental Disorder	2
	Developmental Disorder, Immunological Disease, Cell Cycle	2
	Cellular Development, Lipid Metabolism, Molecular Transport	2
	Cellular Development, Hematological System Development and Function, Hematopoiesis	2

### Validation by qRT-PCR

**Figure 6:**
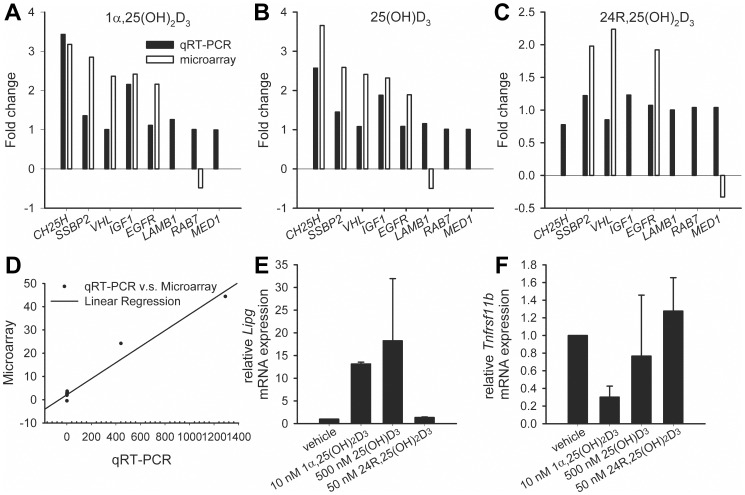
Validation of gene expression by qRT-PCR. (A–C) Gene expression in hP29SN stromal cells. *CH25H*: cholesterol 25-hydroxylase; *SSBP2*: single-stranded DNA binding protein 2; *VHL*: von Hippel-Lindau tumor suppressor; *IGF1*: insulin-like growth factor; *EGFR*: epidermal growth factor receptor; *LAMB1*: laminin subunit β1; *RAB7*: RAB7A, member RAS oncogene family; *MED1*: mediator complex subunit 1. (D) Fold changes of gene expression in hP29SN detected by either qRT-PCR or microarray are plotted and linear regression is shown by a solid line. (E–F) The expression of endothelial lipase (*Lipg*) and tumor necrosis factor receptor superfamily, member 11b (*Tnfrsf11b*) in m*Cyp27b1* ^−/−^ fibroblasts was measured by qRT-PCR. Randomly selected two sets of experiments were pooled to generate final two sets of RNA samples for both microarray and qRT-PCR. Results are expressed as means ± SD.

To validate the microarray data by an independent method, the expression levels of eight genes in hP29SN stromal cells were measured by qRT-PCR (Figure 6A–C). A good correlation (R^2^ = 0.96) was detected between the two methods (Figure 6D). Similarly, the expression of two genes in m*Cyp27b1* ^−/−^ fibroblasts was measured by qRT-PCR (Figure 6E–F).

## Discussion

In order to analyze putative differential gene regulatory effects of vitamin D_3_ metabolites, we performed cDNA microarray analysis in hP29SN stromal cells. To rule out the effect of intracellularly 25(OH)D_3_-derived 1α,25(OH)_2_D_3_, we also performed cDNA microarray analysis in m*Cyp27b1* ^−/−^ fibroblasts that lack 25(OH)D_3_ 1α-hydroxylase enzyme activity.

The optimal serum concentrations of 25(OH)D_3_ for good health are still under discussion. The proposed normal range is broad and variable in different populations perhaps due to an adaptation. Several optimal concentrations have been proposed: 20–80 nM [31], 50–250 nM [32], and 40–70 nM [9] have been suggested. The international recommended optimal concentration of 25(OH)D_3_ is 50 nM or more. 40 nM or more is recommended in Finland, 50 nM by the Institute of Medicine, 75–185 nM (30–74 ng/ml) by MedlinePlus, and 100 nM by the Endocrine Society. On the other hand, the normal serum concentration of 1α,25(OH)_2_D_3_ is clearly defined, being 0.03–0.14 nM [31]. The concentration of 24R,25(OH)_2_D_3_ is approximately 1.44–8 nM (0.6–2.9 ng/ml) [33]. The prefect experimental setup would be using physiological concentrations of metabolites; however, it has been shown that the metabolites at physiological concentrations are not active in cell studies. To avoid bias in comparison and ensure detectable responses, we have chosen 10-fold higher concentrations of vitamin D metabolites over their normal serum levels, which are approximately 50, 5, and 1 nM for 25(OH)D_3_, 24R,25(OH)_2_D_3_, and 1α,25(OH)_2_D_3_, respectively.

Here we identified 1α,25(OH)_2_D_3_ regulated genes involved in immune functions, antibacterial response, and inflammation (Figure 4 and Table 1) as well as DNA repair, cell growth and death, and cell-matrix interaction (Tables S1, S3, and 3), which have been previously described in other cell systems [34]–[36].

### 25OHD_3_ is an Active Hormone

Our study in m*Cyp27b1* ^−/−^ fibroblasts suggests that gene expression fingerprints of 1α,25(OH)_2_D_3_ and 25OHD_3_ are similar. Among the genes regulated by 1α,25(OH)_2_D_3_ in m*Cyp27b1* ^−/−^ fibroblasts, 52% were also regulated by 25(OH)D_3_. On the other hand, 68% of 25(OH)D_3_-regulated genes in m*Cyp27b1* ^−/−^ fibroblasts were also regulated by 1α,25(OH)_2_D_3_. The majority of overlapping genes are the most strongly regulated genes among the top 15 in the lists. These data once further support previous findings by us [3], [4] and others [6]–[8], [10], [12] that 25(OH)D_3_ is indeed an active hormone. Studies of 25(OH)D_3_ analog that is hardly subjected to 1α-hydroxylation provide additional evidence that 1α-hydroxylation of 25(OH)D_3_ is not required for its anti-proliferative activity [12].

Human P29SN stromal cells express 25(OH)D_3_ 1α-hydroxylase that converts 25(OH)D_3_ into 1α,25(OH)_2_D_3_ and vitamin D_3_ 24-hydroxylase that converts 25(OH)D_3_ into 24R,25(OH)_2_D_3_ [3], thus we could not rule out the effects of intracellular 1α,25(OH)_2_D_3_ and 24R,25(OH)_2_D_3_ products derived from 25OHD_3_. We observed that only a few genes commonly regulated by 25(OH)D_3_ and 1α,25(OH)_2_D_3_ in hP29SN. Of the genes regulated by one metabolite, only 12–13% were also regulated by another metabolite. *CYP24A1* was the most highly up-regulated gene by 25(OH)D_3_ and 1α,25(OH)_2_D_3_ in both mouse and human fibroblasts. It is worth mentioning that 24R,25(OH)_2_D_3_ did not regulate *CYP24A1* gene expression. An earlier microarray study has also found the induction ratio of *CYP24A1* gene expression by 1α,25(OH)_2_D_3_ was the highest among 3800 human genes examined and 24R,25(OH)_2_D_3_ at a very high concentration (1000 nM) did induce *CYP24A1* gene expression [37]. The expression of cholesterol 25-hydroxylase was increased more than threefold by both 25(OH)D_3_ and 1α,25(OH)_2_D_3_ in hP29SN, consistent with our previous study [38]. Both 25(OH)D_3_ and 1α,25(OH)_2_D_3_ increased the expression of insulin-like growth factor 1 more than twofold in hP29SN.

These data indeed demonstrate unique roles for 25(OH)D_3_ and 1α,25(OH)_2_D_3_. Recent molecular dynamics simulations data [4] indicate both 1α,25(OH)_2_D_3_ and 25(OH)D_3_ bind to the vitamin D receptor (*VDR*) ligand-binding pocket at the same position, resulting in identical agonistic conformation [4]. Microarray data presented here further validate the biological significance of 25(OH)D_3_.

### A Complementary Role of 25(OH)D_3_ and 1α,25(OH)_2_D_3_ in Calcium and Skeletal Homeostasis

1α,25(OH)_2_D_3_ stimulates a number of genes involved in bone formation [39], [40] and osteoclast differentiation [41]–[43]. We discovered several new 1α,25(OH)_2_D_3_-target genes involved in bone physiology and pathology in hP29SN stromal cells and m*Cyp27b1* ^−/−^ fibroblasts. Na^+^/H^+^ exchanger domain containing 2 (*NHEDC2*, *NHA2*, *SLC9B2*), a member of Na^+^/H^+^ exchanger family, was potently up-regulated by 1α,25(OH)_2_D_3_ (ca. tenfold) in hP29SN stromal cells. Thus, it is tempting to link 1α,25(OH)_2_D_3_ action to bone resorption and remodeling based on a recently-discovered role of *NHEDC2* in osteoclast differentiation and fusion [44], [45]. Sex determining region Y-box 6 (*SOX6*), an osteoporosis susceptibility gene shown by genome-wide association studies [46], [47], was up-regulated by more than twofold by 1α,25(OH)_2_D_3_ in hP29SN. *Sox6* was shown to be essential for cartilage formation in mice [48].

Contrary to human cells, we identified a different set of bone-related genes as the targets of 1α,25(OH)_2_D_3_ in m*Cyp27b1* ^−/−^ fibroblasts. Secreted frizzled related protein 1 (*Sfrp1*) was down-regulated by 1α,25(OH)_2_D_3_. *Sfrp1* was previously shown to inhibit receptor activator of nuclear factor-κB ligand-dependent osteoclast formation [49] and was strongly associated with bone mineral density and bone mineral content [50]. Loss of *Sfrp1* in mice improves fracture repair [51], due to antagonism of the *Wnt* pathway. Bone morphogenetic protein 1 (*BMP1*) was increased by more than sevenfold by 1α,25(OH)_2_D_3_ in m*Cyp27b1* ^−/−^ fibroblasts. *BMP1* has been suggested to enhance bone repair [52].

25(OH)D_3_ shares some functions with 1α,25(OH)_2_D_3_ in regulating calcium and skeletal homeostasis. Three calcium homeostasis-related genes were regulated by 25(OH)D_3_ and 1α,25(OH)_2_D_3_ in both mouse and human fibroblasts. Voltage-dependent calcium channel γ-4 subunit (*CACNG4*) was up-regulated in hP29SN stromal cells (more than twofold by 25(OH)D_3_ and more than threefold by 1α,25(OH)_2_D_3_). S100 calcium binding protein G (*S100G*, *CABP9K*, encoding calbindin D9K) was dramatically up-regulated in m*Cyp27b1* ^−/−^ fibroblasts (88-fold by 25(OH)D_3_ and 85-fold by 1α,25(OH)_2_D_3_). Prolactin receptor (*PRLR*) gene expression was up-regulated by more than threefold by 25(OH)D_3_ and more than 12-fold 1α,25(OH)_2_D_3_ in m*Cyp27b1* ^−/−^ fibroblasts. *PRLP* was responsible for the growth inhibitory effect of prolactin on prostate cells [53] and recently it was found to participate in calcium homeostasis by multiple mechanisms [54]. Two bone-related genes were regulated by both 25(OH)D_3_ and 1α,25(OH)_2_D_3_ in m*Cyp27b1* ^−/−^ fibroblasts. The expression of secreted frizzled related protein 2 (*Sfrp2*) was strongly down-regulated by both 25(OH)D_3_ (sixfold) and 1α,25(OH)_2_D_3_ (sevenfold) in m*Cyp27b1* ^−/−^ fibroblasts. Sfrp2 is an osteoporosis susceptibility gene associated with bone mineral density [50]. In addition, both 25(OH)D_3_ (more than fourfold) and 1α,25(OH)_2_D_3_ (more than fivefold) also inhibited the expression of tumor necrosis factor receptor superfamily, member 11b (*Tnfrsf11b*, *OPG*, osteoprotegerin) in m*Cyp27b1* ^−/−^ fibroblasts. Osteoprotegerin functions as a negative regulator of osteoclastogenesis and bone resorption [55].

Collectively, studies in human prostate stromal cells and mouse skin fibroblasts suggest that 25(OH)D_3_ and 1α,25(OH)_2_D_3_ complement each other in regulating calcium homeostasis and bone remodeling. The role of 1α,25(OH)_2_D_3_ is broader than that of 25(OH)D_3_. Our current findings support *in vivo* data that show partial rescue of the phenotype of *Cyp27b1* ^−/−^ mice by vitamin D_3_ injection [10]. Although human prostate stromal cells and mouse skin fibroblasts are neither the sites of calcium absorption nor the responsible cells for bone formation, the current finding provides new information with indications that require further studies in intestine and bone cells. The action of 25(OH)D_3_ and 1α,25(OH)_2_D_3_ in calcium homeostasis in stromal cells and fibroblasts may have two meanings. Firstly, the second messenger Ca^2+^ is involved in many cellular processes, such as cell proliferation, cell differentiation, cell-cell and cell-matrix interactions, and cell movement. In fact, the role of 1α,25(OH)_2_D_3_ in calcium homeostasis has been described in a microarray study of LNCaP human prostate cancer cells [56]. Secondly, calcium homeostasis in non-bone tissues may indicate soft tissue calcification, which is also a major concern in vitamin D therapy. This is evidenced by a microarray study of human artery smooth muscle cells [57].

We did not find 24R,25(OH)_2_D_3_-target genes related to skeletal homeostasis in hP29SN stromal cells and m*Cyp27b1* ^−/−^ fibroblasts. This should be verified by *in vitro* bone cell studies. It might also be possible that the previously discovered effect of 24R,25(OH)_2_D_3_ on the bone is non-genomic.

### Non-classical Actions of Vitamin D_3_ Metabolites

To get an in-depth view of the actions of each metabolite, we further analyzed the regulated genes not involved in bone and calcium homeostasis. We discovered 1α,25(OH)_2_D_3_-mediated up-regulation of several genes that negatively regulate cell cycle and growth. Tyrosine kinase, non-receptor, 1 (*TNK1*), up-regulated by more than sixfold in hP29SN, was earlier shown to facilitate tumor necrosis factor α-induced apoptosis [58]. Cdk5 and Abl enzyme substrate 1 (*CABLES1*), up-regulated by more than sixfold in hP29SN, is involved in regulation of the cell cycle. Reduction of *CABLES1* expression was previously observed in colorectal cancers [59]. Microcephalin 1 (*MCPH1*) was up-regulated by more than fourfold in hP29SN. *MCPH1* product acts as a DNA-damage response protein and prevents premature entry into mitosis [60].

The vitamin D endocrine system plays an important role in skin in both ligand-dependent and -independent manners [61]. Vitamin D and the *VDR* regulate a number of genes involved in keratinocyte differentiation and hair follicle cycle. Here, we identified three genes whose expression was specifically regulated by 1α,25(OH)_2_D_3_ in m*Cyp27b1* ^−/−^ fibroblasts. Keratin 17, a key regulatory gene in hair development [62], was strongly induced by 1α,25(OH)_2_D_3_ (more than 13-fold). Cadherin 11, which is expressed in mesenchymal aggregates during hair follicle development [63], was up-regulated by 1α,25(OH)_2_D_3_ (threefold). Transforming growth factor β2, shortening hair cycle by stimulating certain caspases [64], was down-regulated by 1α,25(OH)_2_D_3_ (twofold). These findings provide additional important mechanism by which 1α,25(OH)_2_D_3_ specifically regulates hair development in a *VDR*-dependent manner.

The IPA analysis shows that amyotrophic lateral sclerosis (ALS) signaling is the top canonical pathway of 1α,25(OH)_2_D_3_-mediated genes in hP29SN stromal cells (Table 2). This finding supports the recent hypothesis that vitamin D may delay ALS progression [65]–[68].

A number of genes involved in gene transcription were specifically modulated by 25(OH)D_3_ (Figures 2B and 2C). Nuclear receptor coactivator 2 (*NCOA2*, *SRC2*) was induced by 8-fold, hairy and enhancer of split 1 (*HES1*) by more than twofold, activating transcription factor 6 (*ATF6*) by more than twofold, and nuclear transcription factor Y, γ (*NFYC*, *CBF-C*) by more than twofold in hP29SN stromal cells. *HES1*, a Notch-target gene, is a transcription factor involved in various events during development [69]–[72]. *ATF6* mediates *RUNX2*-dependent osteocalcin expression in osteoblasts [73] and apoptosis in myoblasts [74]. *CBF-C* represses the transactivating activity of Smad2 and Smad3 [75]. Several C2H2-like zinc-finger proteins that are involved in transcriptional regulation, for example, YY2 transcription factor and zinc finger proteins, were also induced by 25(OH)D_3_ in hP29SN.

Previous studies have linked vitamin D deficiency to various inflammatory diseases and suggest a role for vitamin D in immunity [76]. Our current study provides an alternative mechanism of the immunomodulatory property of 25(OH)D_3._ 25(OH)D_3_ induced the expression of membrane-associated ring finger (C3HC4) 1 (*MARCH1*) more than sixfold in hP29SN stromal cells. *MARCH1* is an E3 ubiquitin ligase that down-regulates major histocompatibility complex class I membrane expression [77]. The immunosuppressive effect of interleukin-10 is mediated by *MARCH1* [78]. Furthermore, 25(OH)D_3_ increased SAM domain and HD domain 1 (*SAMHD1*) expression more than twofold in hP29SN. *SAMHD1* plays a role in the innate immune response and may regulate tumor necrosis factor α proinflammatory responses [79]. Hepatitis A virus cellular receptor 2 (*HAVCR2*, *TIM3*, T cell immunoglobulin-3) gene expression was increased threefold by 25(OH)D_3_ in hP29SN. *TIM3* fails to be up-regulated in T cells in human autoimmune diseases, such as multiple sclerosis [80]. *TIM3* locus and specific gene polymorphisms are associated with various immune-mediated diseases, such as rheumatoid arthritis [81] and atopic disease [82]. These findings are primary but important. Confirmation utilizing monocytes, dendritic cells, and T cells for *in vitro* studies, together with functional analyses, will bolster the importance of our conclusions.

24R,25(OH)_2_D_3_ is produced by *CYP24A1* when there is enough active vitamin D_3_ available. We identified two 24R,25(OH)_2_D_3_-target genes involved in methylation and epigenetics in hP29SN stromal cells. *MLL5* (a trithorax homolog, myeloid/lymphoid or mixed-lineage leukaemia 5) was up-regulated more than threefold by 24R,25(OH)_2_D_3_. *MLL5* is a known chromatin regulator in H3K4 methylation [83]. Methyltransferase like 15 (*METTL15*, *METT5D1*) was increased more than 15-fold by 24R,25(OH)_2_D_3_.

Human microarray data show that 24R,25(OH)_2_D_3_ modulate cell differentiation and proliferation, in particular by strongly influencing breast cancer 1, early onset (*BRCA1*) signaling pathway (Figure 2D). *BRCA1* expression was increased more than twofold by 24R,25(OH)_2_D_3_. Induction of *BRCA1* was previously found to be partially responsible for the anti-proliferative effects of 1α,25(OH)_2_D_3_ [84]. Interestingly, cofactor of *BRCA1* (*COBRA1*) was reduced threefold by 24R,25(OH)_2_D_3_. *BRCA1* and *COBRA1* seem to function in coordinate gene regulatory pathways [85]. Tumor protein p53 binding protein 1 (*TP53BP1*) was up-regulated by more than threefold by 24R,25(OH)_2_D_3_. *TP53BP1* up-regulates the promoter of *BRCA1* [86]. Kinase suppressor of ras 2 (*KSR2*), a target of 1α,25(OH)_2_D_3_ [87], was increased by more than fivefold by 24R,25(OH)_2_D_3_ in hP29SN. 24R,25(OH)_2_D_3_ regulates two hormone receptor coactivators. It decreased the expression of mediator complex subunit 1 (*MED1*, *TRAP220*, *DRIP205*) threefold. *MED1* is a VDR coactivator [88]. It also decreased thyroid hormone receptor associated protein 3 (twofold).

24R,25(OH)_2_D_3_ influences the expression of several membrane proteins. Erythrocyte membrane protein band 4.1 like 5 (*EPB4.1L5*), which contributes to the correct positioning of tight junction during the establishment of cell polarity [89], was increased ninefold by 24R,25(OH)_2_D_3_ in hP29SNstromal cells. Synaptotagmin XIV (*SYT14*) was up-regulated more than sixfold in hP29SN. Regulating synaptic membrane exocytosis 2 (*Rims2*) was up-regulated more than twofold in m*Cyp27b1* ^−/−^ fibroblasts. Both the gene products are involved in exocytosis.

## Conclusions

The present study is the first systematic comparison of global gene expression in hP29SN stromal cells and m*Cyp27b1* ^−/−^ fibroblasts after treatment with 1α,25(OH)_2_D_3_, 25(OH)D_3_, and 24R,25(OH)_2_D_3._ It seems that there are three partially different vitamin D_3_ (cholecalcipherol) hormonal systems. 25(OH)D_3_ and 1α,25(OH)_2_D_3_ complement each other in regulating calcium homeostasis and bone remodeling. Each vitamin D_3_ metabolite has unique non-classical actions in various physiological and pathological processes. Our study presents a novel perspective for the function of vitamin D_3_ within the endocrine system, and furthers our understanding of the roles and relationships between vitamin D_3_ metabolites. The limitation of our study is that some regulated genes need to be clarified in relevant cells and tissues, e.g. immune responsive genes and bone-related genes.

## Supporting Information

Figure S1 **Quantitative real-time RT-PCR (qRT-PCR) analysis of** ***CYP24A1*** **gene expression.** Human P29SN stromal cells were treated with either 0.1% ethanol (vehicle), 10 nM 1α,25(OH)_2_D_3_, 500 nM 25(OH)D_3_, or 25 nM 24R,25(OH)_2_D_3_ for 24 h. Relative mRNA expression was normalized to the control gene *RPLP0*, and fold inductions were calculated in reference to vehicle. Results are expressed as means ± SD (n = 4). The same samples were then used in microarray assays.
(TIF)

Table S1 **Sequences of the primers used in qPCR.**
(PDF)

Table S2 **Enriched gene ontology (GO) categories for exclusively regulated genes in human P29SN stromal cells generated by DAVID.**
(PDF)

Table S3 **Enriched gene ontology (GO) categories for exclusively regulated genes in mouse** ***Cyp27b1*** **^−/−^ fibroblasts generated by DAVID.**
(PDF)

Table S4 **Enriched gene ontology (GO) categories for commonly regulated genes by both 1α,25(OH)_2_D_3_ and 25(OH)D_3_ in mouse** ***Cyp27b1*** **^−/−^ fibroblasts generated by DAVID.**
(PDF)

## References

[1] Adams JS (2005) “Bound” to work: the free hormone hypothesis revisited. Cell 122: 647–649. 16143095 10.1016/j.cell.2005.08.024

[2] Nykjaer A, Dragun D, Walther D, Vorum H, Jacobsen C, et al (1999) An endocytic pathway essential for renal uptake and activation of the steroid 25-(OH) vitamin D3. Cell 96: 507–515. 10052453 10.1016/s0092-8674(00)80655-8

[3] Lou YR, Laaksi I, Syvala H, Blauer M, Tammela TL, et al (2004) 25-hydroxyvitamin D3 is an active hormone in human primary prostatic stromal cells. FASEB J 18: 332–334. 14657005 10.1096/fj.03-0140fje

[4] Lou YR, Molnar F, Perakyla M, Qiao S, Kalueff AV, et al (2010) 25-Hydroxyvitamin D(3) is an agonistic vitamin D receptor ligand. J Steroid Biochem Mol Biol 118: 162–170. 19944755 10.1016/j.jsbmb.2009.11.011

[5] Lou YR, Tuohimaa P (2006) Androgen enhances the antiproliferative activity of vitamin D3 by suppressing 24-hydroxylase expression in LNCaP cells. J Steroid Biochem Mol Biol 99: 44–49. 16524720 10.1016/j.jsbmb.2005.11.006

[6] Peng X, Hawthorne M, Vaishnav A, St-Arnaud R, Mehta RG (2009) 25-Hydroxyvitamin D3 is a natural chemopreventive agent against carcinogen induced precancerous lesions in mouse mammary gland organ culture. Breast Cancer Res Treat 113: 31–41. 18205042 10.1007/s10549-008-9900-0 PMC2695979

[7] Peng X, Vaishnav A, Murillo G, Alimirah F, Torres KE, et al (2010) Protection against cellular stress by 25-hydroxyvitamin D3 in breast epithelial cells. J Cell Biochem 110: 1324–1333. 20564226 10.1002/jcb.22646

[8] Ritter CS, Armbrecht HJ, Slatopolsky E, Brown AJ (2006) 25-Hydroxyvitamin D(3) suppresses PTH synthesis and secretion by bovine parathyroid cells. Kidney Int 70: 654–659. 16807549 10.1038/sj.ki.5000394

[9] Tuohimaa P, Lou YR (2012) Optimal serum calcidiol concentration for cancer prevention. Anticancer Res 32: 373–381. 22213329

[10] Zhang ZL, Ding XF, Tong J, Li BY (2011) Partial rescue of the phenotype in 1alpha-hydroxylase gene knockout mice by vitamin D3 injection. Endocr Res 36: 101–108. 21329450 10.3109/07435800.2010.542415

[11] Ritter CS, Brown AJ (2011) Direct suppression of Pth gene expression by the vitamin D prohormones doxercalciferol and calcidiol requires the vitamin D receptor. J Mol Endocrinol 46: 63–66. 21169421 10.1677/JME-10-0128

[12] Munetsuna E, Nakabayashi S, Kawanami R, Yasuda K, Ohta M, et al (2011) Mechanism of the anti-proliferative action of 25-hydroxy-19-nor-vitamin D(3) in human prostate cells. J Mol Endocrinol 47: 209–218. 21693624 10.1530/JME-11-0008

[13] Diesel B, Radermacher J, Bureik M, Bernhardt R, Seifert M, et al (2005) Vitamin D(3) metabolism in human glioblastoma multiforme: functionality of CYP27B1 splice variants, metabolism of calcidiol, and effect of calcitriol. Clin Cancer Res 11: 5370–5380. 16061850 10.1158/1078-0432.CCR-04-1968

[14] Boyan BD, Sylvia VL, McKinney N, Schwartz Z (2003) Membrane actions of vitamin D metabolites 1alpha,25(OH)2D3 and 24R,25(OH)2D3 are retained in growth plate cartilage cells from vitamin D receptor knockout mice. J Cell Biochem 90: 1207–1223. 14635194 10.1002/jcb.10716

[15] van Driel M, Koedam M, Buurman CJ, Roelse M, Weyts F, et al (2006) Evidence that both 1alpha,25-dihydroxyvitamin D3 and 24-hydroxylated D3 enhance human osteoblast differentiation and mineralization. J Cell Biochem 99: 922–935. 16741965 10.1002/jcb.20875

[16] Barreto AM, Schwartz GG, Woodruff R, Cramer SD (2000) 25-Hydroxyvitamin D3, the prohormone of 1,25-dihydroxyvitamin D3, inhibits the proliferation of primary prostatic epithelial cells. Cancer Epidemiol Biomarkers Prev 9: 265–270. 10750664

[17] van Driel M, Koedam M, Buurman CJ, Hewison M, Chiba H, et al (2006) Evidence for auto/paracrine actions of vitamin D in bone: 1alpha-hydroxylase expression and activity in human bone cells. FASEB J 20: 2417–2419. 17023519 10.1096/fj.06-6374fje

[18] Li J, Byrne ME, Chang E, Jiang Y, Donkin SS, et al (2008) 1alpha,25-Dihydroxyvitamin D hydroxylase in adipocytes. J Steroid Biochem Mol Biol 112: 122–126. 18840526 10.1016/j.jsbmb.2008.09.006 PMC2602794

[19] Laaksi I, Ruohola JP, Tuohimaa P, Auvinen A, Haataja R, et al (2007) An association of serum vitamin D concentrations <40 nmol/L with acute respiratory tract infection in young Finnish men. Am J Clin Nutr 86: 714–717. 17823437 10.1093/ajcn/86.3.714

[20] Giovannucci E, Liu Y, Rimm EB, Hollis BW, Fuchs CS, et al (2006) Prospective study of predictors of vitamin D status and cancer incidence and mortality in men. J Natl Cancer Inst 98: 451–459. 16595781 10.1093/jnci/djj101

[21] Ruohola JP, Laaksi I, Ylikomi T, Haataja R, Mattila VM, et al (2006) Association between serum 25(OH)D concentrations and bone stress fractures in Finnish young men. J Bone Miner Res 21: 1483–1488. 16939407 10.1359/jbmr.060607

[22] Hypponen E, Laara E, Reunanen A, Jarvelin MR, Virtanen SM (2001) Intake of vitamin D and risk of type 1 diabetes: a birth-cohort study. Lancet 358: 1500–1503. 11705562 10.1016/S0140-6736(01)06580-1

[23] Eyles DW, Burne TH, McGrath JJ (2012) Vitamin D, effects on brain development, adult brain function and the links between low levels of vitamin D and neuropsychiatric disease. Front Neuroendocrinol. 10.1016/j.yfrne.2012.07.001 22796576

[24] Porojnicu A, Robsahm TE, Berg JP, Moan J (2007) Season of diagnosis is a predictor of cancer survival. Sun-induced vitamin D may be involved: a possible role of sun-induced Vitamin D. J Steroid Biochem Mol Biol 103: 675–678. 17229569 10.1016/j.jsbmb.2006.12.031

[25] Tuohimaa P, Tenkanen L, Ahonen M, Lumme S, Jellum E, et al (2004) Both high and low levels of blood vitamin D are associated with a higher prostate cancer risk: a longitudinal, nested case-control study in the Nordic countries. Int J Cancer 108: 104–108. 14618623 10.1002/ijc.11375

[26] Durup D, Jorgensen HL, Christensen J, Schwarz P, Heegaard AM, et al (2012) A reverse J-shaped association of all-cause mortality with serum 25-hydroxyvitamin D in general practice: the CopD study. J Clin Endocrinol Metab 97: 2644–2652. 22573406 10.1210/jc.2012-1176

[27] Kriebitzsch C, Verlinden L, Eelen G, Tan BK, Van Camp M, et al (2009) The impact of 1,25(OH)2D3 and its structural analogs on gene expression in cancer cells–a microarray approach. Anticancer Res 29: 3471–3483. 19667141

[28] Dardenne O, Prud’homme J, Arabian A, Glorieux FH, St-Arnaud R (2001) Targeted inactivation of the 25-hydroxyvitamin D(3)-1(alpha)-hydroxylase gene (CYP27B1) creates an animal model of pseudovitamin D-deficiency rickets. Endocrinology 142: 3135–3141. 11416036 10.1210/endo.142.7.8281

[29] Huang da W, Sherman BT, Lempicki RA (2009) Systematic and integrative analysis of large gene lists using DAVID bioinformatics resources. Nat Protoc 4: 44–57. 19131956 10.1038/nprot.2008.211

[30] Huang da W, Sherman BT, Lempicki RA (2009) Bioinformatics enrichment tools: paths toward the comprehensive functional analysis of large gene lists. Nucleic Acids Res 37: 1–13. 19033363 10.1093/nar/gkn923 PMC2615629

[31] Vieth R, Pinto TR, Reen BS, Wong MM (2002) Vitamin D poisoning by table sugar. Lancet 359: 672. 11879864 10.1016/S0140-6736(02)07814-5

[32] Holick MF (2007) Vitamin D deficiency. N Engl J Med 357: 266–281. 17634462 10.1056/NEJMra070553

[33] Seamark DA, Trafford DJ, Makin HL (1980) The estimation of vitamin D and some metabolites in human plasma by mass fragmentography. Clin Chim Acta 106: 51–62. 6250743 10.1016/0009-8981(80)90374-5

[34] Campos LT, Brentani H, Roela RA, Katayama ML, Lima L, et al (2013) Differences in transcriptional effects of 1alpha,25 dihydroxyvitamin D3 on fibroblasts associated to breast carcinomas and from paired normal breast tissues. J Steroid Biochem Mol Biol 133: 12–24. 22939885 10.1016/j.jsbmb.2012.08.002

[35] Krishnan AV, Shinghal R, Raghavachari N, Brooks JD, Peehl DM, et al (2004) Analysis of vitamin D-regulated gene expression in LNCaP human prostate cancer cells using cDNA microarrays. Prostate 59: 243–251. 15042599 10.1002/pros.20006

[36] Tarroni P, Villa I, Mrak E, Zolezzi F, Mattioli M, et al (2012) Microarray analysis of 1,25(OH)(2)D(3) regulated gene expression in human primary osteoblasts. J Cell Biochem 113: 640–649. 21956231 10.1002/jcb.23392

[37] Tashiro K, Abe T, Oue N, Yasui W, Ryoji M (2004) Characterization of vitamin D-mediated induction of the CYP 24 transcription. Mol Cell Endocrinol 226: 27–32. 15489002 10.1016/j.mce.2004.07.012

[38] Wang JH, Tuohimaa P (2006) Regulation of cholesterol 25-hydroxylase expression by vitamin D3 metabolites in human prostate stromal cells. Biochem Biophys Res Commun 345: 720–725. 16696936 10.1016/j.bbrc.2006.04.156

[39] St-Arnaud R, Naja RP (2011) Vitamin D metabolism, cartilage and bone fracture repair. Mol Cell Endocrinol 347: 48–54. 21664253 10.1016/j.mce.2011.05.018

[40] Bell TD, Demay MB, Burnett-Bowie SA (2010) The biology and pathology of vitamin D control in bone. J Cell Biochem 111: 7–13. 20506379 10.1002/jcb.22661 PMC4020510

[41] Bergh JJ, Xu Y, Farach-Carson MC (2004) Osteoprotegerin expression and secretion are regulated by calcium influx through the L-type voltage-sensitive calcium channel. Endocrinology 145: 426–436. 14525906 10.1210/en.2003-0319

[42] Hofbauer LC, Heufelder AE (2001) Role of receptor activator of nuclear factor-kappaB ligand and osteoprotegerin in bone cell biology. J Mol Med (Berl) 79: 243–253. 11485016 10.1007/s001090100226

[43] Palmqvist P, Persson E, Conaway HH, Lerner UH (2002) IL-6, leukemia inhibitory factor, and oncostatin M stimulate bone resorption and regulate the expression of receptor activator of NF-kappa B ligand, osteoprotegerin, and receptor activator of NF-kappa B in mouse calvariae. J Immunol 169: 3353–3362. 12218157 10.4049/jimmunol.169.6.3353

[44] Ha BG, Hong JM, Park JY, Ha MH, Kim TH, et al (2008) Proteomic profile of osteoclast membrane proteins: identification of Na+/H+ exchanger domain containing 2 and its role in osteoclast fusion. Proteomics 8: 2625–2639. 18600791 10.1002/pmic.200701192

[45] Pham L, Purcell P, Morse L, Stashenko P, Battaglino RA (2007) Expression analysis of nha-oc/NHA2: a novel gene selectively expressed in osteoclasts. Gene Expr Patterns 7: 846–851. 17698421 10.1016/j.modgep.2007.07.002 PMC2271150

[46] Liu YZ, Pei YF, Liu JF, Yang F, Guo Y, et al (2009) Powerful bivariate genome-wide association analyses suggest the SOX6 gene influencing both obesity and osteoporosis phenotypes in males. PLoS One 4: e6827. 19714249 10.1371/journal.pone.0006827 PMC2730014

[47] Rivadeneira F, Styrkarsdottir U, Estrada K, Halldorsson BV, Hsu YH, et al (2009) Twenty bone-mineral-density loci identified by large-scale meta-analysis of genome-wide association studies. Nat Genet 41: 1199–1206. 19801982 10.1038/ng.446 PMC2783489

[48] Smits P, Li P, Mandel J, Zhang Z, Deng JM, et al (2001) The transcription factors L-Sox5 and Sox6 are essential for cartilage formation. Dev Cell 1: 277–290. 11702786 10.1016/s1534-5807(01)00003-x

[49] Hausler KD, Horwood NJ, Chuman Y, Fisher JL, Ellis J, et al (2004) Secreted frizzled-related protein-1 inhibits RANKL-dependent osteoclast formation. J Bone Miner Res 19: 1873–1881. 15476588 10.1359/JBMR.040807

[50] Sims AM, Shephard N, Carter K, Doan T, Dowling A, et al (2008) Genetic analyses in a sample of individuals with high or low BMD shows association with multiple Wnt pathway genes. J Bone Miner Res 23: 499–506. 18021006 10.1359/jbmr.071113

[51] Gaur T, Wixted JJ, Hussain S, O’Connell SL, Morgan EF, et al (2009) Secreted frizzled related protein 1 is a target to improve fracture healing. J Cell Physiol 220: 174–181. 19301255 10.1002/jcp.21747 PMC2756719

[52] Grgurevic L, Macek B, Mercep M, Jelic M, Smoljanovic T, et al (2011) Bone morphogenetic protein (BMP)1–3 enhances bone repair. Biochem Biophys Res Commun 408: 25–31. 21453682 10.1016/j.bbrc.2011.03.109

[53] Wu W, Ginsburg E, Vonderhaar BK, Walker AM (2005) S179D prolactin increases vitamin D receptor and p21 through up-regulation of short 1b prolactin receptor in human prostate cancer cells. Cancer Res 65: 7509–7515. 16103106 10.1158/0008-5472.CAN-04-3350

[54] Ajibade DV, Dhawan P, Fechner AJ, Meyer MB, Pike JW, et al (2010) Evidence for a role of prolactin in calcium homeostasis: regulation of intestinal transient receptor potential vanilloid type 6, intestinal calcium absorption, and the 25-hydroxyvitamin D(3) 1alpha hydroxylase gene by prolactin. Endocrinology 151: 2974–2984. 20463051 10.1210/en.2010-0033 PMC2903940

[55] Yasuda H, Shima N, Nakagawa N, Mochizuki SI, Yano K, et al (1998) Identity of osteoclastogenesis inhibitory factor (OCIF) and osteoprotegerin (OPG): a mechanism by which OPG/OCIF inhibits osteoclastogenesis in vitro. Endocrinology 139: 1329–1337. 9492069 10.1210/endo.139.3.5837

[56] Wang WL, Chatterjee N, Chittur SV, Welsh J, Tenniswood MP (2011) Effects of 1alpha,25 dihydroxyvitamin D3 and testosterone on miRNA and mRNA expression in LNCaP cells. Mol Cancer 10: 58. 21592394 10.1186/1476-4598-10-58 PMC3112430

[57] Shalhoub V, Shatzen EM, Ward SC, Young JI, Boedigheimer M, et al (2010) Chondro/osteoblastic and cardiovascular gene modulation in human artery smooth muscle cells that calcify in the presence of phosphate and calcitriol or paricalcitol. J Cell Biochem 111: 911–921. 20665672 10.1002/jcb.22779 PMC3470918

[58] Azoitei N, Brey A, Busch T, Fulda S, Adler G, et al (2007) Thirty-eight-negative kinase 1 (TNK1) facilitates TNFalpha-induced apoptosis by blocking NF-kappaB activation. Oncogene 26: 6536–6545. 17471239 10.1038/sj.onc.1210476

[59] Park do Y, Sakamoto H, Kirley SD, Ogino S, Kawasaki T, et al (2007) The Cables gene on chromosome 18q is silenced by promoter hypermethylation and allelic loss in human colorectal cancer. Am J Pathol 171: 1509–1519. 17982127 10.2353/ajpath.2007.070331 PMC2043512

[60] Alderton GK, Galbiati L, Griffith E, Surinya KH, Neitzel H, et al (2006) Regulation of mitotic entry by microcephalin and its overlap with ATR signalling. Nat Cell Biol 8: 725–733. 16783362 10.1038/ncb1431

[61] Bikle DD (2010) Vitamin D and the skin. J Bone Miner Metab 28: 117–130. 20107849 10.1007/s00774-009-0153-8

[62] McGowan KM, Tong X, Colucci-Guyon E, Langa F, Babinet C, et al (2002) Keratin 17 null mice exhibit age- and strain-dependent alopecia. Genes Dev 16: 1412–1422. 12050118 10.1101/gad.979502 PMC186322

[63] Nanba D, Nakanishi Y, Hieda Y (2003) Establishment of cadherin-based intercellular junctions in the dermal papilla of the developing hair follicle. Anat Rec A Discov Mol Cell Evol Biol 270: 97–102. 12524684 10.1002/ar.a.10012

[64] Hibino T, Nishiyama T (2004) Role of TGF-beta2 in the human hair cycle. J Dermatol Sci 35: 9–18. 15194142 10.1016/j.jdermsci.2003.12.003

[65] Solomon JA, Gianforcaro A, Hamadeh MJ (2011) Vitamin D3 deficiency differentially affects functional and disease outcomes in the G93A mouse model of amyotrophic lateral sclerosis. PLoS One 6: e29354. 22216257 10.1371/journal.pone.0029354 PMC3246470

[66] Sato Y, Honda Y, Asoh T, Kikuyama M, Oizumi K (1997) Hypovitaminosis D and decreased bone mineral density in amyotrophic lateral sclerosis. Eur Neurol 37: 225–229. 9208262 10.1159/000117447

[67] Shen L (2011) Further support for vitamin D supplement in delaying the progression of ALS. Med Hypotheses 77: 698. 10.1016/j.mehy.2011.07.057 21855226

[68] Karam C, Scelsa SN (2011) Can vitamin D delay the progression of ALS? Med Hypotheses 76: 643–645. 21310542 10.1016/j.mehy.2011.01.021

[69] Ishibashi M, Ang SL, Shiota K, Nakanishi S, Kageyama R, et al (1995) Targeted disruption of mammalian hairy and Enhancer of split homolog-1 (HES-1) leads to up-regulation of neural helix-loop-helix factors, premature neurogenesis, and severe neural tube defects. Genes Dev 9: 3136–3148. 8543157 10.1101/gad.9.24.3136

[70] Kopinke D, Brailsford M, Shea JE, Leavitt R, Scaife CL, et al (2011) Lineage tracing reveals the dynamic contribution of Hes1+ cells to the developing and adult pancreas. Development 138: 431–441. 21205788 10.1242/dev.053843 PMC3014632

[71] Monahan P, Rybak S, Raetzman LT (2009) The notch target gene HES1 regulates cell cycle inhibitor expression in the developing pituitary. Endocrinology 150: 4386–4394. 19541765 10.1210/en.2009-0206 PMC2736073

[72] Shibata K, Yamada H, Sato T, Dejima T, Nakamura M, et al (2011) Notch-Hes1 pathway is required for the development of IL-17-producing gammadelta T cells. Blood 118: 586–593. 21606479 10.1182/blood-2011-02-334995

[73] Jang WG, Kim EJ, Kim DK, Ryoo HM, Lee KB, et al (2012) BMP2 protein regulates osteocalcin expression via Runx2-mediated Atf6 gene transcription. J Biol Chem 287: 905–915. 22102412 10.1074/jbc.M111.253187 PMC3256879

[74] Morishima N, Nakanishi K, Nakano A (2011) Activating transcription factor-6 (ATF6) mediates apoptosis with reduction of myeloid cell leukemia sequence 1 (Mcl-1) protein via induction of WW domain binding protein 1. J Biol Chem 286: 35227–35235. 21841196 10.1074/jbc.M111.233502 PMC3186435

[75] Chen F, Ogawa K, Liu X, Stringfield TM, Chen Y (2002) Repression of Smad2 and Smad3 transactivating activity by association with a novel splice variant of CCAAT-binding factor C subunit. Biochem J 364: 571–577. 12023901 10.1042/BJ20011703 PMC1222603

[76] Adams JS, Hewison M (2008) Unexpected actions of vitamin D: new perspectives on the regulation of innate and adaptive immunity. Nat Clin Pract Endocrinol Metab 4: 80–90. 18212810 10.1038/ncpendmet0716 PMC2678245

[77] Bartee E, Mansouri M, Hovey Nerenberg BT, Gouveia K, Fruh K (2004) Downregulation of major histocompatibility complex class I by human ubiquitin ligases related to viral immune evasion proteins. J Virol 78: 1109–1120. 14722266 10.1128/JVI.78.3.1109-1120.2004 PMC321412

[78] Thibodeau J, Bourgeois-Daigneault MC, Huppe G, Tremblay J, Aumont A, et al (2008) Interleukin-10-induced MARCH1 mediates intracellular sequestration of MHC class II in monocytes. Eur J Immunol 38: 1225–1230. 18389477 10.1002/eji.200737902 PMC2759377

[79] Liao W, Bao Z, Cheng C, Mok YK, Wong WS (2008) Dendritic cell-derived interferon-gamma-induced protein mediates tumor necrosis factor-alpha stimulation of human lung fibroblasts. Proteomics 8: 2640–2650. 18546154 10.1002/pmic.200700954

[80] Koguchi K, Anderson DE, Yang L, O’Connor KC, Kuchroo VK, et al (2006) Dysregulated T cell expression of TIM3 in multiple sclerosis. J Exp Med 203: 1413–1418. 16754722 10.1084/jem.20060210 PMC2118310

[81] Chae SC, Park YR, Shim SC, Yoon KS, Chung HT (2004) The polymorphisms of Th1 cell surface gene Tim-3 are associated in a Korean population with rheumatoid arthritis. Immunol Lett 95: 91–95. 15325803 10.1016/j.imlet.2004.06.008

[82] Chae SC, Park YR, Lee YC, Lee JH, Chung HT (2004) The association of TIM-3 gene polymorphism with atopic disease in Korean population. Hum Immunol 65: 1427–1431. 15603868 10.1016/j.humimm.2004.07.002

[83] Sebastian S, Sreenivas P, Sambasivan R, Cheedipudi S, Kandalla P, et al (2009) MLL5, a trithorax homolog, indirectly regulates H3K4 methylation, represses cyclin A2 expression, and promotes myogenic differentiation. Proc Natl Acad Sci U S A 106: 4719–4724. 19264965 10.1073/pnas.0807136106 PMC2651835

[84] Campbell MJ, Gombart AF, Kwok SH, Park S, Koeffler HP (2000) The anti-proliferative effects of 1alpha,25(OH)2D3 on breast and prostate cancer cells are associated with induction of BRCA1 gene expression. Oncogene 19: 5091–5097. 11042697 10.1038/sj.onc.1203888

[85] Aiyar SE, Cho H, Lee J, Li R (2007) Concerted transcriptional regulation by BRCA1 and COBRA1 in breast cancer cells. Int J Biol Sci 3: 486–492. 18071589 10.7150/ijbs.3.486 PMC2096739

[86] Rauch T, Zhong X, Pfeifer GP, Xu X (2005) 53BP1 is a positive regulator of the BRCA1 promoter. Cell Cycle 4: 1078–1083. 15970701

[87] Wang X, Wang TT, White JH, Studzinski GP (2007) Expression of human kinase suppressor of Ras 2 (hKSR-2) gene in HL60 leukemia cells is directly upregulated by 1,25-dihydroxyvitamin D(3) and is required for optimal cell differentiation. Exp Cell Res 313: 3034–3045. 17599832 10.1016/j.yexcr.2007.05.021 PMC3351793

[88] Ren Y, Behre E, Ren Z, Zhang J, Wang Q, et al (2000) Specific structural motifs determine TRAP220 interactions with nuclear hormone receptors. Mol Cell Biol 20: 5433–5446. 10891484 10.1128/mcb.20.15.5433-5446.2000 PMC85995

[89] Gosens I, Sessa A, den Hollander AI, Letteboer SJ, Belloni V, et al (2007) FERM protein EPB41L5 is a novel member of the mammalian CRB-MPP5 polarity complex. Exp Cell Res 313: 3959–3970. 17920587 10.1016/j.yexcr.2007.08.025

