# Correction: Gene Expression Profiles in Human and Mouse Primary Cells Provide New Insights into the Differential Actions of Vitamin D_3_ Metabolites

**DOI:** 10.1371/annotation/9cb2000b-a962-453c-ad8b-088f91095f6d

**Published:** 2013-11-15

**Authors:** Pentti Tuohimaa, Jing-Huan Wang, Sofia Khan, Marianne Kuuslahti, Kui Qian, Tommi Manninen, Petri Auvinen, Mauno Vihinen, Yan-Ru Lou

In the Author Contributions Statement, the second author (JHW) should be noted as one of the authors who conceived and designed the experiments, and the list of authors who performed the experiments should read in the following order: JHW MK YRL TM.

In addition, there were multiple errors in the Results. The eighth sentence of the third paragraph of the "Gene Expression Profiles Induced by Vitamin D

3 Metabolites" section of the Results should read: "To

understand the specific role of each vitamin D3 metabolite, we grouped the regulated genes into commonly and individually regulated gene groups (Figure 1C)." The first sentence of the second paragraph of the "Functional Annotation of Genes Regulated by Vitamin D3 Metabolites" section of the Results should read: In hP29SN stromal cells, the highly enriched GO categories related to 1a, 25(OH)2D3-treatment include those involved in nucleotide biosynthesis, ubiquitin conjugation, ion binding, and macromolecule biosynthesis (Table S2). 25(OH)D3 plays numerous roles in intracellular organelles, DNA-binding transcription factors, protein-protein interaction, transcription coregulator activity, RNA-binding motif, and organic acid biosynthesis (Table S2). 

