# Supplementary figures and images for: Gene Expression Profiles in Human and Mouse Primary Cells Provide New Insights into the Differential Actions of Vitamin D3 Metabolites
Source: PLoS One. 2013 Oct 8;8(10):e75338. doi: 10.1371/journal.pone.0075338 (PMC3792969; doi:10.1371/journal.pone.0075338)

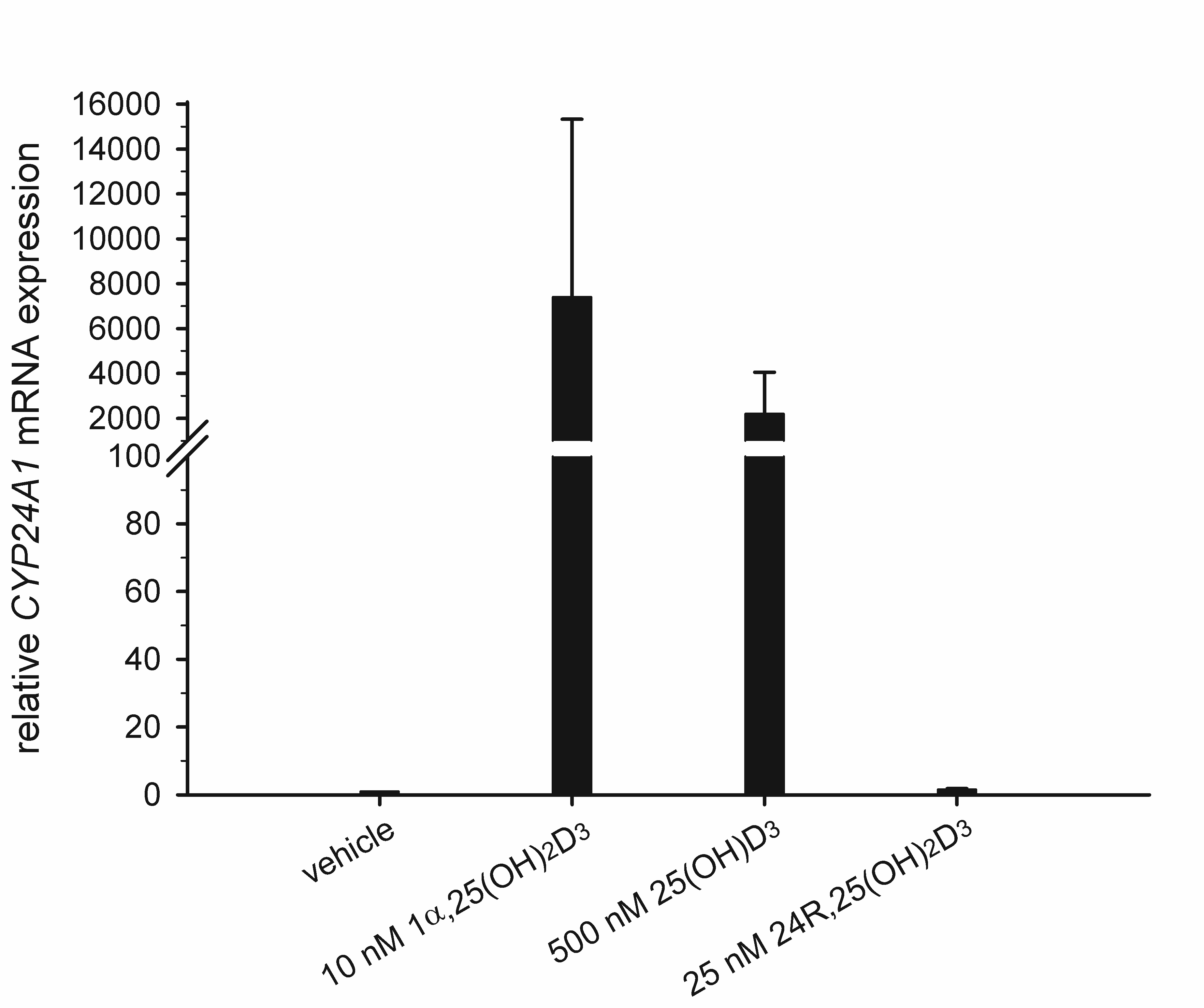

Supplement: Figure S1 — Quantitative real-time RT-PCR (qRT-PCR) analysis of CYP24A1 gene expression. Human P29SN stromal cells were treated with either 0.1% ethanol (vehicle), 10 nM 1α,25(OH)2D3, 500 nM 25(OH)D3, or 25 nM 24R,25(OH)2D3 for 24 h. Relative mRNA expression was normalized to the control gene RPLP0, and fold inductions were calculated in reference to vehicle. Results are expressed as means ± SD (n = 4). The same samples were then used in microarray assays. (TIF) [file pone.0075338.s001.tif]
