# Supplementary material for: Gene Expression Profiles in Human and Mouse Primary Cells Provide New Insights into the Differential Actions of Vitamin D3 Metabolites
Source: PLoS One. 2013 Oct 8;8(10):e75338. doi: 10.1371/journal.pone.0075338 (PMC3792969; doi:10.1371/journal.pone.0075338)
Supplement: Table S1 — Sequences of the primers used in qPCR. (PDF) [file pone.0075338.s002.pdf]

Table S1. Sequences of the primers used in qPCR.

Gene or EST name	Primer sequence	Position
RPLP0	Forward 5’-AATCTCCAGGGGCACCATT-3’	515-533
NM_001002	Reverse 5’-CGCTGGCTCCCACTTTGT-3’	588-571
CYP24A1	Forward 5’-GCCCAGCCGGGAACTC-3’	1907-1922
NM_000782	Reverse 5’-AAATACCACCATCTGAGGCGTATT-3’	1968-1945
CH25H	Forward 5’-TCCTGTTCTGCCTGCTACTCTTC-3’	405-427
NM_003956	Reverse 5’-GGTACAGCCAGGGCACCTT-3’	482-464
VHL	Forward 5’- AGAGCGATGCCTCCAGGTT-3’	690-708
NM_000551	Reverse 5’- TGACGATGTCCAGTCTCCTGTAA-3’	757-735
SSBP2	Forward 5’-GGAGGCAGGAAAGTCCAAGAT-3’	
BC041991	Reverse 5’-ATGTGAGGATGCTGCAAGAAAG-3’	
IGF1	Forward 5’- GGCATAGCTGGCCAAACAA-3’	4389-4371
NM_001111283	Reverse 5’- CACTTGGGAGAAGGCTTAGAATAAA-3’	4317-4341
EGFR	Forward 5’- GCGTCTCTTGCCGGAATGT-3’	1814-1832
NM_005228	Reverse 5’- GGCTCACCCTCCAGAAGGTT-3’	1883-1864
LAMB1	Forward 5’- AAACCAAATCTTATCTCTTTGAGACTCA-3’	
N30158	Reverse 5’- TCTGCAAAATATAGTGCCTTCGAA-3’	
RAB7	Forward 5’- TGATGGTGGATGACAGGCTAGT-3’	381-402
NM_004637	Reverse 5’- CGAGAGACTGGAACCGTTCCT-3’	452-432
MED1	Forward 5’-GAGACAGTTGAAGACATGGTGAAAAA-3’	1800-1825
NM_004774	Reverse 5’- GCCTGTGGTCATGCCATACC-3’	1871-1852
Tbp	Forward Primer: 5’-CTTCGTGCAAGAAATGCTGAATAT-3’	490-513
NM_013684	Reverse Primer: 5’-CCGTGGCTCTCTTATTCTCATGA-3’	561-539
Lipg	Forward Primer: 5’-CAGGCTGTGGATTCAATGATGT-3’	1038-1059
NM_010720	Reverse Primer: 5’-TCGCATTTCACCATCTCTGAGA-3’	1107-1086
Tnfrsf11b	Forward Primer: 5’-TCCCGAGGACCACAATGAAC-3’	224-243
NM_008764	Reverse Primer: 5’-TCCTGGGTTGTCCATTCAATG-3’	302-282

RPLP0: acidic ribosomal phosphoprotein P0; *CYP24A1*: 24-hydroxylase; *CH25H*: cholesterol 25-hydroxylase; *VHL*: von Hippel-Lindau tumor suppressor; *SSBP2*: single-stranded DNA binding protein 2; *IGF1*: insulin-like growth factor; *EGFR*: epidermal growth factor receptor; *LAMB1*: laminin subunit β1; *RAB7*: RAB7A, member RAS oncogene family; *MED1*: mediator complex subunit 1; *Tbp*: TATA box binding protein; *Lipg*: endothelial lipase; *Tnfrsf11b*: tumor necrosis factor receptor superfamily, member 11b (osteoprotegerin).
