# Supplementary material for: Gene Expression Profiles in Human and Mouse Primary Cells Provide New Insights into the Differential Actions of Vitamin D3 Metabolites
Source: PLoS One. 2013 Oct 8;8(10):e75338. doi: 10.1371/journal.pone.0075338 (PMC3792969; doi:10.1371/journal.pone.0075338)
Supplement: Table S2 — Enriched gene ontology (GO) categories for exclusively regulated genes in human P29SN stromal cells generated by DAVID. (PDF) [file pone.0075338.s003.pdf]

Table S2. Enriched gene ontology (GO) categories for exclusively regulated genes in human P29SN stromal cells generated by DAVID.

**Genes regulated only by 10 nM 1α,25(OH)$_2$D$_3$**		
Category	Term	*p*-Value
Annotation Cluster 2	*Enrichment Score: 1.0526103311151749*	
GOTERM_BP_FAT	GO:0006259~DNA metabolic process	0.010
Annotation Cluster 3	*Enrichment Score: 0.9336547388058266*	
SP_PIR_KEYWORDS	ubl conjugation pathway	0.040
Annotation Cluster 7	*Enrichment Score: 0.7466633006717093*	
GOTERM_MF_FAT	GO:0031420~alkali metal ion binding	0.021
Annotation Cluster 8	*Enrichment Score: 0.6832284096027084*	
GOTERM_MF_FAT	GO:0043167~ion binding	0.016
GOTERM_MF_FAT	GO:0046872~metal ion binding	0.022
GOTERM_MF_FAT	GO:0043169~cation binding	0.025
Annotation Cluster 9	*Enrichment Score: 0.6799962081523407*	
GOTERM_BP_FAT	GO:0010605~negative regulation of macromolecule metabolic process	0.022
GOTERM_BP_FAT	GO:0010558~negative regulation of macromolecule biosynthetic process	0.044
GOTERM_BP_FAT	GO:0031327~negative regulation of cellular biosynthetic process	0.049

**Genes regulated only by 500 nM 25(OH)D$_3$**		
Category	Term	*p*-Value
Annotation Cluster 1	*Enrichment Score: 2.0177809714626043*	
GOTERM_CC_FAT	GO:0070013~intracellular organelle lumen	0.004
GOTERM_CC_FAT	GO:0043233~organelle lumen	0.005
GOTERM_CC_FAT	GO:0031974~membrane-enclosed lumen	0.006
GOTERM_CC_FAT	GO:0005654~nucleoplasm	0.009
GOTERM_CC_FAT	GO:0031981~nuclear lumen	0.012
Annotation Cluster 2	*Enrichment Score: 1.7860151666805686*	
GOTERM_BP_FAT	GO:0051252~regulation of RNA metabolic process	0.003
GOTERM_BP_FAT	GO:0006357~regulation of transcription from RNA polymerase II promoter	0.003
INTERPRO	IPR007087:Zinc finger, C2H2-type	0.005
INTERPRO	IPR015880:Zinc finger, C2H2-like	0.006
GOTERM_BP_FAT	GO:0006355~regulation of transcription, DNA-dependent	0.006
GOTERM_MF_FAT	GO:0030528~transcription regulator activity	0.009
SP_PIR_KEYWORDS	nucleus	0.009
SMART	SM00355:ZnF_C2H2	0.016
SP_PIR_KEYWORDS	transcription regulation	0.017
SP_PIR_KEYWORDS	dna-binding	0.017
SP_PIR_KEYWORDS	Transcription	0.020

GOTERM_BP_FAT	GO:0045449~regulation of transcription	0.028
GOTERM_BP_FAT	GO:0006350~transcription	0.028
INTERPRO	IPR013087:Zinc finger, C2H2-type/integrase, DNA-binding	0.033
Annotation Cluster 3	*Enrichment Score: 1.5573464838687392*	
UP_SEQ_FEATURE	repeat:ANK 3	0.015
UP_SEQ_FEATURE	repeat:ANK 1	0.026
UP_SEQ_FEATURE	repeat:ANK 2	0.027
SP_PIR_KEYWORDS	ank repeat	0.029
INTERPRO	IPR002110:Ankyrin	0.030
SMART	SM00248:ANK	0.050
Annotation Cluster 4	*Enrichment Score: 1.5429055231838762*	
SP_PIR_KEYWORDS	zinc-finger	0.003
INTERPRO	IPR007087:Zinc finger, C2H2-type	0.005
INTERPRO	IPR015880:Zinc finger, C2H2-like	0.006
SP_PIR_KEYWORDS	metal-binding	0.013
SMART	SM00355:ZnF_C2H2	0.016
GOTERM_MF_FAT	GO:0046914~transition metal ion binding	0.020
SP_PIR_KEYWORDS	zinc	0.032
INTERPRO	IPR013087:Zinc finger, C2H2-type/integrase, DNA-binding	0.033
Annotation Cluster 5	*Enrichment Score: 1.3280446589611756*	
GOTERM_BP_FAT	GO:0006357~regulation of transcription from RNA polymerase II promoter	0.003
GOTERM_BP_FAT	GO:0006355~regulation of transcription, DNA-dependent	0.006
GOTERM_CC_FAT	GO:0005654~nucleoplasm	0.009
GOTERM_MF_FAT	GO:0030528~transcription regulator activity	0.009
GOTERM_MF_FAT	GO:0016563~transcription activator activity	0.019
GOTERM_BP_FAT	GO:0045941~positive regulation of transcription	0.023
GOTERM_MF_FAT	GO:0003713~transcription coactivator activity	0.025
GOTERM_BP_FAT	GO:0010628~positive regulation of gene expression	0.026
GOTERM_BP_FAT	GO:0045893~positive regulation of transcription, DNA-dependent	0.032
GOTERM_BP_FAT	GO:0051254~positive regulation of RNA metabolic process	0.034
GOTERM_BP_FAT	GO:0030518~steroid hormone receptor signaling pathway	0.034
GOTERM_BP_FAT	GO:0045935~positive regulation of nucleobase, nucleoside, nucleotide and nucleic acid metabolic process	0.037
GOTERM_MF_FAT	GO:0003712~transcription cofactor activity	0.039
GOTERM_BP_FAT	GO:0051173~positive regulation of nitrogen compound metabolic process	0.042
GOTERM_BP_FAT	GO:0010557~positive regulation of macromolecule biosynthetic process	0.045
Annotation Cluster 6	*Enrichment Score: 1.2251280654849863*	
GOTERM_BP_FAT	GO:0006357~regulation of transcription from RNA polymerase II promoter	0.003
GOTERM_MF_FAT	GO:0016564~transcription repressor activity	0.006

GOTERM_MF_FAT	GO:0030528~transcription regulator activity	0.009
SP_PIR_KEYWORDS	repressor	0.019
GOTERM_BP_FAT	GO:0016481~negative regulation of transcription	0.028
GOTERM_BP_FAT	GO:0030902~hindbrain development	0.037
GOTERM_BP_FAT	GO:0010629~negative regulation of gene expression	0.041
GOTERM_BP_FAT	GO:0045934~negative regulation of nucleobase, nucleoside, nucleotide and nucleic acid metabolic process	0.043
GOTERM_BP_FAT	GO:0000122~negative regulation of transcription from RNA polymerase II promoter	0.045
GOTERM_BP_FAT	GO:0051172~negative regulation of nitrogen compound metabolic process	0.046
Annotation Cluster 7	*Enrichment Score: 1.1399468539342168*	
UP_SEQ_FEATURE	domain:RRM 1	0.013
UP_SEQ_FEATURE	domain:RRM 2	0.013
Annotation Cluster 8	*Enrichment Score: 0.9238015216303497*	
INTERPRO	IPR007087:Zinc finger, C2H2-type	0.005
INTERPRO	IPR015880:Zinc finger, C2H2-like	0.006
SMART	SM00355:ZnF_C2H2	0.016
INTERPRO	IPR013087:Zinc finger, C2H2-type/integrase, DNA-binding	0.033
Annotation Cluster 9	*Enrichment Score: 0.8145515801861937*	
GOTERM_BP_FAT	GO:0046394~carboxylic acid biosynthetic process	0.043
GOTERM_BP_FAT	GO:0016053~organic acid biosynthetic process	0.043

Genes regulated only by 25 nM 24R,25(OH)$_2$D$_3$

Category	Term	*p*-Value
Annotation Cluster 1	*Enrichment Score: 1.9702677999826337*	
SP_PIR_KEYWORDS	zinc-finger	0.000
SP_PIR_KEYWORDS	metal-binding	0.000
SP_PIR_KEYWORDS	Zinc	0.002
GOTERM_MF_FAT	GO:0008270~zinc ion binding	0.023
GOTERM_MF_FAT	GO:0046914~transition metal ion binding	0.036
Annotation Cluster 2	*Enrichment Score: 1.7506703521909222*	
GOTERM_BP_FAT	GO:0043414~biopolymer methylation	0.009
GOTERM_BP_FAT	GO:0032259~methylation	0.011
GOTERM_BP_FAT	GO:0006479~protein amino acid methylation	0.024
GOTERM_BP_FAT	GO:0008213~protein amino acid alkylation	0.024
GOTERM_BP_FAT	GO:0006730~one-carbon metabolic process	0.031
Annotation Cluster 3	*Enrichment Score: 1.5215974026427899*	
SP_PIR_KEYWORDS	Nucleus	0.003
SP_PIR_KEYWORDS	Transcription	0.016
GOTERM_BP_FAT	GO:0045449~regulation of transcription	0.021
SP_PIR_KEYWORDS	transcription regulation	0.025
GOTERM_BP_FAT	GO:0006350~transcription	0.027

GOTERM_BP_FAT	GO:0006355~regulation of transcription, DNA-dependent	0.039
GOTERM_BP_FAT	GO:0051252~regulation of RNA metabolic process	0.047
Annotation Cluster 4	*Enrichment Score: 1.4998334589406268*	
GOTERM_CC_FAT	GO:0044454~nuclear chromosome part	0.004
GOTERM_CC_FAT	GO:0000228~nuclear chromosome	0.011
GOTERM_CC_FAT	GO:0005694~chromosome	0.012
GOTERM_CC_FAT	GO:0044427~chromosomal part	0.018
SP_PIR_KEYWORDS	cell cycle	0.018
GOTERM_BP_FAT	GO:0033554~cellular response to stress	0.023
GOTERM_CC_FAT	GO:0000785~chromatin	0.023
SP_PIR_KEYWORDS	dna repair	0.026
GOTERM_BP_FAT	GO:0006974~response to DNA damage stimulus	0.027
SP_PIR_KEYWORDS	DNA damage	0.032
GOTERM_CC_FAT	GO:0000793~condensed chromosome	0.032
GOTERM_CC_FAT	GO:0000790~nuclear chromatin	0.035
GOTERM_MF_FAT	GO:0003684~damaged DNA binding	0.047
GOTERM_BP_FAT	GO:0007049~cell cycle	0.048
Annotation Cluster 5	*Enrichment Score: 1.4860327874110117*	
INTERPRO	IPR008271:Serine/threonine protein kinase, active site	0.001
INTERPRO	IPR002290:Serine/threonine protein kinase	0.004
INTERPRO	IPR017442:Serine/threonine protein kinase-related	0.005
UP_SEQ_FEATURE	active site:Proton acceptor	0.005
UP_SEQ_FEATURE	domain:Protein kinase	0.006
SP_PIR_KEYWORDS	serine/threonine-protein kinase	0.007
INTERPRO	IPR000719:Protein kinase, core	0.007
GOTERM_MF_FAT	GO:0004674~protein serine/threonine kinase activity	0.010
SMART	SM00220:S_TKc	0.010
UP_SEQ_FEATURE	nucleotide phosphate-binding region:ATP	0.011
SP_PIR_KEYWORDS	transferase	0.015
GOTERM_BP_FAT	GO:0006793~phosphorus metabolic process	0.015
GOTERM_BP_FAT	GO:0006796~phosphate metabolic process	0.015
INTERPRO	IPR017441:Protein kinase, ATP binding site	0.018
GOTERM_BP_FAT	GO:0006468~protein amino acid phosphorylation	0.021
SP_PIR_KEYWORDS	atp-binding	0.022
GOTERM_MF_FAT	GO:0004672~protein kinase activity	0.024
SP_PIR_KEYWORDS	nucleotide-binding	0.035
UP_SEQ_FEATURE	binding site:ATP	0.040
SP_PIR_KEYWORDS	serine/threonine-specific protein kinase	0.048
Annotation Cluster 6	*Enrichment Score: 1.4065466647064622*	
INTERPRO	IPR019786:Zinc finger, PHD-type, conserved site	0.002
UP_SEQ_FEATURE	zinc finger region:PHD-type 1	0.016
INTERPRO	IPR001965:Zinc finger, PHD-type	0.016
SMART	SM00249:PHD	0.026

GOTERM_BP_FAT	GO:0051276~chromosome organization	0.029
SP_PIR_KEYWORDS	chromatin regulator	0.036
SP_PIR_KEYWORDS	repressor	0.042
Annotation Cluster 7	*Enrichment Score: 1.3257604022723697*	
GOTERM_CC_FAT	GO:0019898~extrinsic to membrane	0.017
UP_SEQ_FEATURE	metal ion-binding site:Calcium 2; via carbonyl oxygen	0.022
UP_SEQ_FEATURE	metal ion-binding site:Calcium 2	0.041
Annotation Cluster 8	*Enrichment Score: 1.289696018519006*	
INTERPRO	IPR018029:C2 membrane targeting protein	0.025
INTERPRO	IPR000008:C2 calcium-dependent membrane targeting	0.047
Annotation Cluster 9	*Enrichment Score: 1.235493407917891*	
INTERPRO	IPR001841:Zinc finger, RING-type	0.008
SMART	SM00184:RING	0.019
Annotation Cluster 10	*Enrichment Score: 1.220128163077948*	
INTERPRO	IPR017442:Serine/threonine protein kinase-related	0.005
