# Supplementary material for: Gene Expression Profiles in Human and Mouse Primary Cells Provide New Insights into the Differential Actions of Vitamin D3 Metabolites
Source: PLoS One. 2013 Oct 8;8(10):e75338. doi: 10.1371/journal.pone.0075338 (PMC3792969; doi:10.1371/journal.pone.0075338)
Supplement: Table S3 — Enriched gene ontology (GO) categories for exclusively regulated genes in mouse Cyp27b1 −/− fibroblasts generated by DAVID. (PDF) [file pone.0075338.s004.pdf]

Table S3. Enriched gene ontology (GO) categories for exclusively regulated genes in mouse *Cyp27b1*$^{-/-}$ fibroblasts generated by DAVID.

**Genes regulated only by 10 nM 1α,25(OH)$_2$D$_3$**		
Category	Term	*p*-Value
Annotation Cluster 1	*Enrichment Score: 3.5276665078108884*	
GOTERM_BP_FAT	GO:0045597~positive regulation of cell differentiation	0.000
GOTERM_BP_FAT	GO:0051094~positive regulation of developmental process	0.000
GOTERM_BP_FAT	GO:0045596~negative regulation of cell differentiation	0.019
Annotation Cluster 2	*Enrichment Score: 3.160346739696052*	
SP_PIR_KEYWORDS	glycoprotein	0.000
UP_SEQ_FEATURE	glycosylation site:N-linked (GlcNAc...)	0.000
GOTERM_CC_FAT	GO:0005578~proteinaceous extracellular matrix	0.000
GOTERM_CC_FAT	GO:0031012~extracellular matrix	0.000
GOTERM_CC_FAT	GO:0044421~extracellular region part	0.000
SP_PIR_KEYWORDS	signal	0.000
SP_PIR_KEYWORDS	disulfide bond	0.001
UP_SEQ_FEATURE	signal peptide	0.001
UP_SEQ_FEATURE	disulfide bond	0.003
SP_PIR_KEYWORDS	Secreted	0.004
GOTERM_CC_FAT	GO:0044420~extracellular matrix part	0.005
GOTERM_CC_FAT	GO:0005576~extracellular region	0.007
GOTERM_CC_FAT	GO:0005604~basement membrane	0.011
GOTERM_CC_FAT	GO:0005615~extracellular space	0.017
SP_PIR_KEYWORDS	extracellular matrix	0.037
Annotation Cluster 3	*Enrichment Score: 3.010135248587642*	
GOTERM_BP_FAT	GO:0007155~cell adhesion	0.000
GOTERM_BP_FAT	GO:0022610~biological adhesion	0.000
SP_PIR_KEYWORDS	cell adhesion	0.013
Annotation Cluster 4	*Enrichment Score: 2.949105380780214*	
GOTERM_BP_FAT	GO:0030850~prostate gland development	0.000
GOTERM_BP_FAT	GO:0001655~urogenital system development	0.000
GOTERM_BP_FAT	GO:0060429~epithelium development	0.000
GOTERM_BP_FAT	GO:0022612~gland morphogenesis	0.000
GOTERM_BP_FAT	GO:0048732~gland development	0.000
GOTERM_BP_FAT	GO:0002009~morphogenesis of an epithelium	0.001
GOTERM_BP_FAT	GO:0048729~tissue morphogenesis	0.002
GOTERM_BP_FAT	GO:0060740~prostate gland epithelium morphogenesis	0.003
GOTERM_BP_FAT	GO:0060512~prostate gland morphogenesis	0.004
GOTERM_BP_FAT	GO:0048608~reproductive structure development	0.004
GOTERM_BP_FAT	GO:0003006~reproductive developmental process	0.034
Annotation Cluster 5	*Enrichment Score: 2.4817395458018425*	
GOTERM_MF_FAT	GO:0008083~growth factor activity	0.000

SP_PIR_KEYWORDS	growth factor	0.000
KEGG_PATHWAY	mmu04060:Cytokine-cytokine receptor interaction	0.003
GOTERM_CC_FAT	GO:0005615~extracellular space	0.017
Annotation Cluster 6	*Enrichment Score: 2.1471383765536878*	
GOTERM_MF_FAT	GO:0008083~growth factor activity	0.000
SP_PIR_KEYWORDS	mitogen	0.000
GOTERM_BP_FAT	GO:0051781~positive regulation of cell division	0.000
GOTERM_BP_FAT	GO:0051302~regulation of cell division	0.000
SP_PIR_KEYWORDS	growth factor	0.000
KEGG_PATHWAY	mmu05211:Renal cell carcinoma	0.002
INTERPRO	IPR000072:Platelet-derived growth factor (PDGF)	0.004
SMART	SM00141:PDGF	0.007
GOTERM_BP_FAT	GO:0006928~cell motion	0.010
GOTERM_BP_FAT	GO:0016477~cell migration	0.021
KEGG_PATHWAY	mmu04510:Focal adhesion	0.033
GOTERM_BP_FAT	GO:0048870~cell motility	0.047
GOTERM_BP_FAT	GO:0051674~localization of cell	0.047
Annotation Cluster 7	*Enrichment Score: 2.0167470310432103*	
GOTERM_BP_FAT	GO:0002053~positive regulation of mesenchymal cell proliferation	0.002
GOTERM_BP_FAT	GO:0010464~regulation of mesenchymal cell proliferation	0.002
GOTERM_BP_FAT	GO:0050679~positive regulation of epithelial cell proliferation	0.005
GOTERM_BP_FAT	GO:0009967~positive regulation of signal transduction	0.015
GOTERM_BP_FAT	GO:0050678~regulation of epithelial cell proliferation	0.036
Annotation Cluster 8	*Enrichment Score: 2.012273926877482*	
GOTERM_BP_FAT	GO:0048666~neuron development	0.000
GOTERM_BP_FAT	GO:0030182~neuron differentiation	0.000
GOTERM_BP_FAT	GO:0030030~cell projection organization	0.001
GOTERM_BP_FAT	GO:0031175~neuron projection development	0.004
GOTERM_BP_FAT	GO:0032989~cellular component morphogenesis	0.008
GOTERM_BP_FAT	GO:0048858~cell projection morphogenesis	0.009
GOTERM_BP_FAT	GO:0000902~cell morphogenesis	0.010
GOTERM_BP_FAT	GO:0006928~cell motion	0.010
GOTERM_BP_FAT	GO:0000904~cell morphogenesis involved in differentiation	0.012
GOTERM_BP_FAT	GO:0032990~cell part morphogenesis	0.012
GOTERM_BP_FAT	GO:0021955~central nervous system neuron axonogenesis	0.013
GOTERM_BP_FAT	GO:0048812~neuron projection morphogenesis	0.016
GOTERM_BP_FAT	GO:0048667~cell morphogenesis involved in neuron differentiation	0.019
GOTERM_BP_FAT	GO:0007409~axonogenesis	0.040
Annotation Cluster 9	*Enrichment Score: 1.9787023556258587*	
UP_SEQ_FEATURE	domain:EGF-like 9	0.000

UP_SEQ_FEATURE	domain:EGF-like 8	0.000
UP_SEQ_FEATURE	domain:EGF-like 13	0.001
UP_SEQ_FEATURE	domain:EGF-like 10	0.001
UP_SEQ_FEATURE	domain:EGF-like 6	0.001
UP_SEQ_FEATURE	domain:EGF-like 2	0.003
UP_SEQ_FEATURE	domain:EGF-like 7	0.003
UP_SEQ_FEATURE	domain:EGF-like 12	0.006
UP_SEQ_FEATURE	domain:EGF-like 3	0.009
UP_SEQ_FEATURE	domain:EGF-like 15	0.009
UP_SEQ_FEATURE	domain:EGF-like 14	0.011
UP_SEQ_FEATURE	domain:EGF-like 5	0.012
INTERPRO	IPR006209:EGF	0.014
SP_PIR_KEYWORDS	egf-like domain	0.015
INTERPRO	IPR000152:EGF-type aspartate/asparagine hydroxylation conserved site	0.020
INTERPRO	IPR013032:EGF-like region, conserved site	0.021
INTERPRO	IPR000742:EGF-like, type 3	0.023
UP_SEQ_FEATURE	domain:EGF-like 4	0.024
INTERPRO	IPR006210:EGF-like	0.025
Annotation Cluster 10	*Enrichment Score: 1.7655756290309987*	
GOTERM_BP_FAT	GO:0022612~gland morphogenesis	0.000
GOTERM_BP_FAT	GO:0048732~gland development	0.000
GOTERM_BP_FAT	GO:0001763~morphogenesis of a branching structure	0.003
GOTERM_BP_FAT	GO:0030879~mammary gland development	0.018
GOTERM_BP_FAT	GO:0048754~branching morphogenesis of a tube	0.023
GOTERM_BP_FAT	GO:0060603~mammary gland duct morphogenesis	0.036

Genes regulated only by 500 nM 25(OH)D_3

Category	Term	*p*-Value
Annotation Cluster 1	*Enrichment Score: 1.8054593879563023*	
GOTERM_BP_FAT	GO:0006915~apoptosis	0.003
GOTERM_BP_FAT	GO:0012501~programmed cell death	0.003
GOTERM_BP_FAT	GO:0008219~cell death	0.005
GOTERM_BP_FAT	GO:0016265~death	0.005
SP_PIR_KEYWORDS	Apoptosis	0.006
Annotation Cluster 2	*Enrichment Score: 1.6087166995340825*	
GOTERM_BP_FAT	GO:0010876~lipid localization	0.009
GOTERM_BP_FAT	GO:0006869~lipid transport	0.040
SP_PIR_KEYWORDS	lipid transport	0.043
Annotation Cluster 3	*Enrichment Score: 1.589423219748234*	
SP_PIR_KEYWORDS	glycoprotein	0.010
SP_PIR_KEYWORDS	disulfide bond	0.013
UP_SEQ_FEATURE	glycosylation site:N-linked (GlcNAc...)	0.020

SP_PIR_KEYWORDS	signal	0.021
GOTERM_CC_FAT	GO:0005576~extracellular region	0.035
SP_PIR_KEYWORDS	Secreted	0.039
UP_SEQ_FEATURE	disulfide bond	0.042
Annotation Cluster 4	*Enrichment Score: 1.2660938408638478*	
GOTERM_MF_FAT	GO:0008201~heparin binding	0.013
GOTERM_MF_FAT	GO:0005539~glycosaminoglycan binding	0.030
GOTERM_CC_FAT	GO:0044421~extracellular region part	0.038
GOTERM_MF_FAT	GO:0001871~pattern binding	0.041
GOTERM_MF_FAT	GO:0030247~polysaccharide binding	0.041
Annotation Cluster 5	*Enrichment Score: 1.1640220270630979*	
GOTERM_BP_FAT	GO:0048514~blood vessel morphogenesis	0.008
GOTERM_BP_FAT	GO:0001974~blood vessel remodeling	0.008
GOTERM_BP_FAT	GO:0001568~blood vessel development	0.019
GOTERM_BP_FAT	GO:0001944~vasculature development	0.021
GOTERM_BP_FAT	GO:0048771~tissue remodeling	0.036
Annotation Cluster 6	*Enrichment Score: 1.0727238300317519*	
GOTERM_BP_FAT	GO:0008283~cell proliferation	0.005
Annotation Cluster 7	*Enrichment Score: 1.057856731672389*	
GOTERM_BP_FAT	GO:0008283~cell proliferation	0.005
GOTERM_BP_FAT	GO:0048584~positive regulation of response to stimulus	0.030
Annotation Cluster 9	*Enrichment Score: 0.9412425657474854*	
GOTERM_BP_FAT	GO:0009611~response to wounding	0.006
Annotation Cluster 10	*Enrichment Score: 0.9351872979790068*	
GOTERM_BP_FAT	GO:0008283~cell proliferation	0.005
GOTERM_BP_FAT	GO:0040007~growth	0.034

Genes regulated only by 50 nM 24R,25$(OH)_2D_3$

Category	Term	*p*-Value
Annotation Cluster 2	*Enrichment Score: 0.9369333459647183*	
GOTERM_MF_FAT	GO:0046872~metal ion binding	0.043
GOTERM_MF_FAT	GO:0043169~cation binding	0.046
GOTERM_MF_FAT	GO:0043167~ion binding	0.050
