# Supplementary material for: Gene Expression Profiles in Human and Mouse Primary Cells Provide New Insights into the Differential Actions of Vitamin D3 Metabolites
Source: PLoS One. 2013 Oct 8;8(10):e75338. doi: 10.1371/journal.pone.0075338 (PMC3792969; doi:10.1371/journal.pone.0075338)
Supplement: Table S4 — Enriched gene ontology (GO) categories for commonly regulated genes by both 1α,25(OH)2D3 and 25(OH)D3 in mouse Cyp27b1 −/− fibroblasts generated by DAVID. (PDF) [file pone.0075338.s005.pdf]

Table S4. Enriched gene ontology (GO) categories for commonly regulated genes by both 1α,25$(OH)_2D_3$ and 25$(OH)D_3$ in mouse *Cyp27b1*$^{-/-}$ fibroblasts generated by DAVID.

**Genes regulated by both 10 nM 1α,25$(OH)_2D_3$ and 500 nM 25$(OH)D_3$**		
Category	Term	*p*-Value
Annotation Cluster 1	Enrichment Score: 12.950718485029892	
SP_PIR_KEYWORDS	glycoprotein	0.000
SP_PIR_KEYWORDS	signal	0.000
SP_PIR_KEYWORDS	disulfide bond	0.000
UP_SEQ_FEATURE	glycosylation site:N-linked (GlcNAc...)	0.000
SP_PIR_KEYWORDS	Secreted	0.000
UP_SEQ_FEATURE	disulfide bond	0.000
UP_SEQ_FEATURE	signal peptide	0.000
GOTERM_CC_FAT	GO:0005576~extracellular region	0.000
Annotation Cluster 2	Enrichment Score: 3.3579824639576437	
GOTERM_CC_FAT	GO:0005578~proteinaceous extracellular matrix	0.000
GOTERM_CC_FAT	GO:0031012~extracellular matrix	0.000
SP_PIR_KEYWORDS	collagen degradation	0.000
GOTERM_BP_FAT	GO:0030574~collagen catabolic process	0.000
GOTERM_BP_FAT	GO:0044243~multicellular organismal catabolic process	0.000
GOTERM_BP_FAT	GO:0032963~collagen metabolic process	0.000
GOTERM_BP_FAT	GO:0044259~multicellular organismal macromolecule metabolic process	0.000
GOTERM_MF_FAT	GO:0004222~metalloendopeptidase activity	0.000
GOTERM_BP_FAT	GO:0044236~multicellular organismal metabolic process	0.000
INTERPRO	IPR016293:Peptidase M10A, matrix metallopeotidase	0.000
INTERPRO	IPR006025:Peptidase M, neutral zinc metallopeptidases, zinc-binding site	0.000
INTERPRO	IPR002477:Peptidoglycan binding-like	0.000
PIR_SUPERFAMILY	PIRSF001191:Peptidase_M10A_matrix	0.000
GOTERM_MF_FAT	GO:0008237~metallopeptidase activity	0.000
INTERPRO	IPR000585:Hemopexin/matrixin	0.000
INTERPRO	IPR018486:Hemopexin/matrixin, conserved site	0.000
INTERPRO	IPR018487:Hemopexin/matrixin, repeat	0.000
UP_SEQ_FEATURE	domain:Hemopexin-like 3	0.000
UP_SEQ_FEATURE	domain:Hemopexin-like 4	0.000
GOTERM_MF_FAT	GO:0004175~endopeptidase activity	0.000
UP_SEQ_FEATURE	domain:Hemopexin-like 1	0.000
UP_SEQ_FEATURE	domain:Hemopexin-like 2	0.000
SMART	SM00120:HX	0.000
SP_PIR_KEYWORDS	extracellular matrix	0.000
INTERPRO	IPR006026:Peptidase, metallopeptidases	0.000
SMART	SM00235:ZnMc	0.000

PIR_SUPERFAMILY	PIRSF001191:matrix metalloproteinase, stromelysin type	0.000
INTERPRO	IPR001818:Peptidase M10A and M12B, matrixin and adamalysin	0.001
GOTERM_MF_FAT	GO:0070011~peptidase activity, acting on L-amino acid peptides	0.001
GOTERM_MF_FAT	GO:0008233~peptidase activity	0.002
UP_SEQ_FEATURE	short sequence motif:Cysteine switch	0.002
SP_PIR_KEYWORDS	metalloprotease	0.002
UP_SEQ_FEATURE	metal ion-binding site:Calcium 3; via carbonyl oxygen	0.003
UP_SEQ_FEATURE	propeptide:Activation peptide	0.004
SP_PIR_KEYWORDS	metalloproteinase	0.007
UP_SEQ_FEATURE	metal ion-binding site:Zinc 2; in inhibited form	0.008
SP_PIR_KEYWORDS	zymogen	0.011
UP_SEQ_FEATURE	metal ion-binding site:Calcium 2; via carbonyl oxygen	0.014
GOTERM_BP_FAT	GO:0006508~proteolysis	0.014
UP_SEQ_FEATURE	metal ion-binding site:Zinc 2; catalytic	0.015
UP_SEQ_FEATURE	metal ion-binding site:Zinc; catalytic	0.022
UP_SEQ_FEATURE	metal ion-binding site:Calcium 1	0.023
UP_SEQ_FEATURE	metal ion-binding site:Calcium 2	0.040
SP_PIR_KEYWORDS	Protease	0.045
Annotation Cluster 3	Enrichment Score: 3.24116791784555	
INTERPRO	IPR018097:EGF-like calcium-binding, conserved site	0.000
INTERPRO	IPR006210:EGF-like	0.000
INTERPRO	IPR001881:EGF-like calcium-binding	0.000
INTERPRO	IPR000152:EGF-type aspartate/asparagine hydroxylation conserved site	0.000
INTERPRO	IPR006209:EGF	0.000
SMART	SM00181:EGF	0.000
SMART	SM00179:EGF_CA	0.000
INTERPRO	IPR013032:EGF-like region, conserved site	0.000
SP_PIR_KEYWORDS	egf-like domain	0.000
INTERPRO	IPR000742:EGF-like, type 3	0.000
UP_SEQ_FEATURE	domain:EGF-like 6	0.002
UP_SEQ_FEATURE	domain:EGF-like 1	0.003
UP_SEQ_FEATURE	domain:EGF-like 2	0.004
UP_SEQ_FEATURE	domain:EGF-like 4	0.005
SP_PIR_KEYWORDS	notch signaling pathway	0.011
UP_SEQ_FEATURE	domain:EGF-like 3	0.012
UP_SEQ_FEATURE	domain:EGF-like 5	0.016
INTERPRO	IPR001438:EGF-like, type 2	0.024
GOTERM_BP_FAT	GO:0007219~Notch signaling pathway	0.028
INTERPRO	IPR013091:EGF calcium-binding	0.046
Annotation Cluster 4	Enrichment Score: 2.4497729172410354	

GOTERM_BP_FAT	GO:0008544~epidermis development	0.000
GOTERM_BP_FAT	GO:0007398~ectoderm development	0.001
GOTERM_BP_FAT	GO:0060429~epithelium development	0.002
GOTERM_BP_FAT	GO:0009913~epidermal cell differentiation	0.003
GOTERM_BP_FAT	GO:0030855~epithelial cell differentiation	0.017
GOTERM_BP_FAT	GO:0030216~keratinocyte differentiation	0.020
Annotation Cluster 5	Enrichment Score: 2.058239137874073	
INTERPRO	IPR000884:Thrombospondin, type 1 repeat	0.000
SMART	SM00209:TSP1	0.001
Annotation Cluster 6	Enrichment Score: 1.949896685297675	
GOTERM_BP_FAT	GO:0018149~peptide cross-linking	0.001
INTERPRO	IPR001102:Transglutaminase, N-terminal	0.004
INTERPRO	IPR013808:Transglutaminase, conserved site	0.004
INTERPRO	IPR008958:Transglutaminase, C-terminal	0.004
GOTERM_MF_FAT	GO:0003810~protein-glutamine gamma-glutamyltransferase activity	0.005
INTERPRO	IPR002931:Transglutaminase-like	0.006
SMART	SM00460:TGc	0.008
GOTERM_MF_FAT	GO:0016755~transferase activity, transferring amino-acyl groups	0.018
UP_SEQ_FEATURE	metal ion-binding site:Calcium	0.050
Annotation Cluster 7	Enrichment Score: 1.9182723957765455	
SP_PIR_KEYWORDS	growth factor	0.000
GOTERM_MF_FAT	GO:0008083~growth factor activity	0.002
KEGG_PATHWAY	mmu04060:Cytokine-cytokine receptor interaction	0.002
GOTERM_CC_FAT	GO:0005615~extracellular space	0.003
SP_PIR_KEYWORDS	cytokine	0.020
GOTERM_MF_FAT	GO:0005125~cytokine activity	0.023
BIOCARTA	m_erythPathway:Erythrocyte Differentiation Pathway	0.023
Annotation Cluster 8	Enrichment Score: 1.721763277599938	
GOTERM_MF_FAT	GO:0043167~ion binding	0.000
GOTERM_MF_FAT	GO:0046872~metal ion binding	0.001
GOTERM_MF_FAT	GO:0043169~cation binding	0.002
SP_PIR_KEYWORDS	metal-binding	0.007
Annotation Cluster 9	Enrichment Score: 1.6587981525703623	
GOTERM_BP_FAT	GO:0007411~axon guidance	0.007
GOTERM_BP_FAT	GO:0000902~cell morphogenesis	0.013
GOTERM_BP_FAT	GO:0006928~cell motion	0.014
GOTERM_BP_FAT	GO:0007409~axonogenesis	0.015
GOTERM_BP_FAT	GO:0000904~cell morphogenesis involved in differentiation	0.015
GOTERM_BP_FAT	GO:0030030~cell projection organization	0.016
GOTERM_BP_FAT	GO:0031175~neuron projection development	0.017
GOTERM_BP_FAT	GO:0048812~neuron projection morphogenesis	0.020

GOTERM_BP_FAT	GO:0048667~cell morphogenesis involved in neuron differentiation	0.024
GOTERM_BP_FAT	GO:0032989~cellular component morphogenesis	0.028
GOTERM_BP_FAT	GO:0048858~cell projection morphogenesis	0.037
GOTERM_BP_FAT	GO:0032990~cell part morphogenesis	0.045
Annotation Cluster 10	Enrichment Score: 1.6264123345827333	
KEGG_PATHWAY	mmu04360:Axon guidance	0.002
INTERPRO	IPR003659:Plexin/semaphorin/integrin	0.016
SMART	SM00423:PSI	0.026
INTERPRO	IPR001627:Semaphorin/CD100 antigen	0.050
